# Comparative analysis of the mitochondrial genomes of *Colletotrichum gloeosporioides* sensu lato: insights into the evolution of a fungal species complex interacting with diverse plants

**DOI:** 10.1186/s12864-016-3480-x

**Published:** 2017-02-15

**Authors:** Xiaofei Liang, Xianglin Tian, Wenkui Liu, Tingyu Wei, Wei Wang, Qiuyue Dong, Bo Wang, Yanan Meng, Rong Zhang, Mark L. Gleason, Guangyu Sun

**Affiliations:** 1 State Key Laboratory of Crop Stress Biology in Arid Areas and College of Plant Protection, Northwest A&F University, Yangling, Shaanxi Province 712100 China; 2 Department of Plant Pathology and Microbiology, Iowa State University, Ames, IA 50011 USA

**Keywords:** Evolution, Species complex, Mitochondrial genome, Concatenation, Phylogeny

## Abstract

**Background:**

The fungal species complex *Colletotrichum gloeosporioides* sensu lato contains over 20 plant-interacting species. These species exhibit different life styles (e.g., endophytes, foliar and fruit pathogens) and show considerable variation in host and tissue adaptation strategies. Accurate species delimitation in *C. gloeosporioides* s.l*.* is very challenging due to nascent lineage boundaries and phenotypic plasticity, which strongly impedes studies of the complex’s host-interaction biology. In this study, we first sequenced and compared nine mitogenomes belonging to four *C. gloeosporioides* s.l*.* species lineages (C. *gloeosporioides, C. fructicola, C. aenigma,* and *C. siamense* s.l.), and evaluated the usefulness of mitogenome sequence in complementing prevailing nuclear markers for species delimitation.

**Results:**

The *C. gloeosporioides* s.l*.* mitogenomes ranged between 52,671 and 58,666 bp in size, and each contained an identical set of genes transcribed in the same direction. Compared with previously reported *Colletotrichum* mitogenomes, these mitogenomes were uniquely featured by: (1) significantly larger genome size due to richer intron content and longer intergenic region; (2) striking GC content elevation at the intergenic region; and (3) considerable intron content variation among different species lineages. Compared with nuclear DNA markers commonly used in phylogeny, the mitogenome nucleotide diversity was extremely low, yet the mitogenome alignment contained the highest number of parsimony informative sites, which allowed the generation of a high-resolution phylogeny recognizing all taxonomic lineages, including ones belonging to the very nascent *C. siamense* s.l*.* complex. The tree topology was highly congruent with the phylogeny based on nuclear marker concatenation except for lineages within *C. siamense* s.l. Further comparative phylogenetic analysis indicated that lineage-specific rapid divergence of GS and SOD2 markers confounded concatenation-based species relationship inference.

**Conclusions:**

This study sheds light on the evolution of *C. gloeosporioides* s.l*.* mitogenomes and demonstrates that mitogenome sequence can complement prevailing nuclear markers in improving species delimitation accuracy. The mitogenome sequences reported will be valuable resources for further genetic studies with *C. gloeosporioides* s.l*.* and other *Colletotrichum* species.

**Electronic supplementary material:**

The online version of this article (doi:10.1186/s12864-016-3480-x) contains supplementary material, which is available to authorized users.

## Background

*Colletotrichum gloeosporioides* sensu lato is a fungal species complex encompassing more than 20 closely-related species [1]. These species interact with diverse herbaceous and woody plants as endophytes or foliar/fruit pathogens, and can be classified as generalists or specialists based on their host range specificity [2]. The diverse life styles within *C. gloeosporioides* s.l. makes it an interesting complex to study plant-microbe interactions and understand how host specificity evolves. As phytopathogens, *C. gloeosporioides* s.l. infect foliar as well as fruit tissues, and can cause severe field disease epidemics such as Glomerella leaf spot disease on apple (*Malus domestica* cv. Gala) and coffee berry disease on Arabica coffee (*Coffea arabica*). While many *C. gloeosporioides* s.l. species are economically deleterious, certain members are economically beneficial, e.g., as biocontrol agents against weeds and phytopathogens [3, 4].

Accurate species recognition in *C. gloeosporioides* s.l. is a complex task. Morphological characters, such as the size and shape of conidia and appressoria, are very plastic, and thus cannot be relied on for precise species identification [1, 5]. The species appear to have diverged relatively recently, so no single molecular marker commonly used in taxonomy (e.g., ITS, actin, β-tubulin 2) is informative enough to distinguish all lineages. Species recognition thus relies heavily on multi-locus phylogeny [6], and on the aid of highly polymorphic markers [7–10].

When the lineage divergence time gets very short, one critical issue for phylogeny-based species recognition is gene tree/species tree discordance due to evolutionary events such as incomplete lineage sorting, recombination, and horizontal transfer [11, 12]. Incomplete lineage sorting, or the inherently stochastic nature of gene genealogies at shallow levels of diversification, in particular, is the most common factor causing tree discordance which confounds species inference [12]; the noise/signal ratio can be strong enough to affect multi-locus phylogeny outcome, where different gene combination schemes produce well-supported yet conflicting topologies, making species inference a tricky process [13, 14]. Without a consensus on the concatenation markers and the criteria to draw species boundary lines, inconsistent species classification is unavoidable [11, 15, 16]. A striking example is *C. siamense* s.l., for which the number of proposed species has ranged from one to seven based on analysis with different marker combinations [1, 15, 17].

Several approaches can be taken to improve the consistency and accuracy of nascent species classifications. First, an increased sample size is helpful in teasing species-level variation apart from population-level ones. Second, different analytical approaches (e.g., concatenation-based phylogeny, genealogical concordance analysis, and coalescence-based methods) can be applied to the same set of data for cross validation purpose. Third, an improvement on the number, representativeness, and independence of analyzed genetic markers can reduce the bias effect of gene/species tree discordance.

Compared with nuclear DNA, mitochondrial DNA (mtDNA) mutate more rapidly due to a higher copy number, a greater exposure to reactive oxygen species, and a less efficient DNA repair system [18]. The mitogenome is also characterized by small size, haploidy, a shortage of recombination, and uniparental heredity. Mitochondrial DNA has been used to resolve evolutionary relationships of a wide range of eukaryotic life lineages at multiple taxonomic levels [19–22]. However, phylogenetic analysis within *C. gloeosporioides* s.l. has been based solely on nuclear DNA markers, whereas the suitability of mitochondrial DNA has not been tested, due in large part to a lack of available mitogenome sequence. It is worth investigating whether mitochondrial DNA, as a cytoplasmic factor independent of the prevailing nuclear markers, can provide additional insights into the genetic relationships of *C. gloeosporioides* s.l. lineages.

The first objective of this study was to obtain and compare mitogenomes from representative *C. gloeosporioides* s.l*.* lineages. Weir et al*.* [1] established Musae and Kahawae as the two main *C. gloeosporioides* s.l. clades, into which most recognized species can be classified. In this study, we analyzed nine isolates from four species lineages within or close to the Musae clade. Sequence comparison identified unique characteristics not observed with any previously reported *Colletotrichum* mitogenomes, including a significant size expansion and a striking GC content elevation at the intergenic regions. When comparing the mitogenomes belonging to the *C. gloeosporioides* s.l*.* complex, we observed considerable intron content variation among otherwise highly conserved mitogenomes. Such intron presence–absence polymorphism pattern can potentially be exploited for a rapid genetic diversity survey. Three introns were uniquely conserved among all *C. gloeosporioides* s.l. mitogenomes, indicating their potential value for developing *C. gloeosporioides* s.l. differentiation markers. We tested four primer pairs targeting these introns, and identified one primer pair that was highly efficient in differentiating *C. gloeosporioides* s.l. from *C. actutatum* s.l..

With complete mitogenome sequences available, we also tested the usefulness of mitogenome-based phylogeny in helping resolving the genetic relationships of *C. gloeosporioides* s.l. lineages. Our results demonstrated that mitogenome-based phylogeny recognizes nascent species divergence and is an important complement to nuclear markers in understanding *C. gloeosporioides* complex evolution.

## Methods

### Fungal isolates

Diseased plant samples used for fungal isolation were collected from different geographic regions in China in accordance with local regulations. Details about the taxonomy, host, locality, and DNA marker accessions of fungal isolates are listed in Additional file 1: Table S1. Isolates used for genome sequencing were isolated from diseased apple (*Malus domestica*) leaf or fruit tissues. *C. fructicola* isolates used for intron content survey were isolated from fruit or leaf lesions of apple, kiwifruit (*Actinidia* spp.), chili pepper (*Capsicum annuum*), pear (*Pyrus* spp.), papaya (*Carica papaya*), and nectarine (*Prunus persica* var. *nucipersica*). All fungal cultures were single-spore purified, maintained on potato dextrose agar (PDA), and stored as glycerol stock (15%) at −80 °C in the Fungal Laboratory of Northwest A&F University, Yangling, Shaanxi Province, China.

### Genome sequencing and assembly

Fungal mycelia were produced and harvested from liquid potato dextrose broth (PDB) shake culture. Total genomic DNA was extracted based on a modified cetyl trimethylammonium bromide (CTAB) protocol [23], which was then used to prepare sequencing libraries with a mean insertion size of 350 bp. The libraries were subjected to 100-bp pair-end sequencing with the Illumina HiSeq 2000 platform at the Novogene Genomic Sequencing Center, Beijing, China. Raw data (raw reads) of fastq format were first processed through in-house perl scripts. In this step, clean data (clean reads) were obtained by removing reads containing adapter, reads contains ploy-N (*N* > 10%), and low-quality reads (sQ < = 5) from raw data. Clean reads were then *de novo* assembled into scaffolds using the AbySS assembler version 1.3.5 [24]. Contigs representing mitochondrial genomic DNAs were identified by BLAST search. Gaps were filled by PCR and standard Sanger sequencing, with the used primers listed in Additional file 1: Table S2.

### Sequence annotation and comparison

Coding genes, introns, novel ORFs, rRNAs, and tRNAs were identified based on analysis with the online server MFannot (http://megasun.bch.umontreal.ca/cgi-bin/mfannot/mfannotInterface.pl), RNAweasel (http://megasun.bch.umontreal.ca/cgi-bin/RNAweasel/RNAweaselInterface.pl), and tRNAscan-SE 1.2.1 [25]. The annotations were manually inspected by comparing with *Colletotrichum* mitogenomes using the Mauve software version 2.4.0 [26]. To predict the functions of identified novel ORFs (mostly homing endonuclease genes), the coding proteins were searched against the NCBI nr and InterProScan databases. All measures of genetic diversity were calculated by DNASP version 5.10.1 [27].

### Phylogenetic analysis

To accurately define the phylogenetic placement of the nine isolates used for mitogenome comparison, a Bayesian phylogenetic analysis was performed, including 33 additional *C. gloeosporioides* s.l. isolates along with two *C. alatae* isolates as outgroup. All 35 isolates were derived from a previous study by Weir et al*.* [1]. Inferences were based on seven combined nuclear loci, including actin (ACT), calmodulin (CAL), chitin synthase (CHS), glyceraldehyde-3-phosphate dehydrogenase (GAPDH), glutamine synthetase (GS), manganese-superoxide dismutase (SOD2), and β-tubulin 2 (TUB2). DNA sequences for the seven loci were obtained from local draft genome assemblies, whereas DNA sequences for the referent isolates were downloaded from the NCBI database (please refer to Weir et al*.* [1] for accession numbers). For each gene, the sequences from different isolates were aligned with MEGA version 7 [28] and manually adjusted if necessary. Individual alignments were concatenated using SequenceMatrix version 1.8 [29]. MrModeltest version 2.3 [30] was used to identify the AIC best-fit nucleotide substitution model for each gene partition, which were GTR + I for GADPH, GTR + G for CAL, HKY for ACT, SYM + I for CHS, GTR + G for GS, HKY + G for TUB2, and HKY + I + G for SOD2. Phylogeny construction was based on Bayesian inference (BI) performed with MrBayes version 3.2.1 [31]. The Bayesian analysis included two separate runs for 5 × 10^7^ generations, each sampled every 1000 generations, convergence of all parameters was checked using the internal diagnostics. To construct the 50% major rule consensus tree, the first 25% generations were discarded as burn-in. The same procedure was used to construct a six-locus phylogeny based on a concatenated alignment excluding GS, and a five-locus phylogeny excluding GS as well as SOD2.

Maximum likelihood phylogenetic trees used for comparative phylogenetic analysis were constructed with MEGA7. Best substitution model for each alignment was identified with either ProtTest version 3.2 (for amino acids) [32] or MrModeltest version 2.3 (for nucleotides). For aligning the whole mitogenome, the Mauve software version 2.4.0 [26] was used to, and the conserved sites were further extracted with Gblocks version 0.91b to obtain the final Gblocks alignment dataset [33]. Shimodaira-Hasegawa (SH) test was performed with the PAUP* test-version 4.0a150 (www.paup.scs.fsu.edu). For each test, the null distribution was generated by 10,000 bootstrap re-sampling via RELL approximation.

### Survey of intron content polymorphism

To survey intron content diversity in *C. fructicola*, 14 isolates in addition to 1104–7 were selected (Additional file 1: Table S1). The species identity of these isolates was confirmed by *ApMAT*-locus sequencing. In total, 10 intron positions within five mitochondrial genes (*cob*, *cox1*, *nad1*, *cox3*, *nad2*, *nad5*) were surveyed. Primer pairs (Additional file 1: Table S2) were designed so that for a specific intron position, PCR amplicons of different sizes would be obtained between isolates containing a given intron and isolates lacking that intron. Four primer pairs targeting the three introns that were uniquely conserved among all *C. gloeosporioides* s.l. mitogenomes (cob-1, cox1-4, and cox1-5) were designed and their amplification efficiency and specificity were tested with a set of *C. gloeosporioides* s.l. and *C. actutatum* isolates (Isolates listed in Additional file 1: Table S1; primer sequences listed in Additional file 1: Table S2). Primer designs were based on sequence alignments of all known *Colletotrichum* mitogenomes. The PCRs were performed in a Bio-Rad thermal cycler in a total volume of 25 μL. The PCR mixtures contained 19 μL of double deionized water, 2.5 μL of 10 × PCR buffer (with 20 mM MgCl_2_), 0.5 μL of dNTPs (each 10 mM), 1 μL of each primer (10 μM), 1 μL of genomic DNA, and 0.2 μL (1U) of *EasyTaq* DNA polymerase (Transgen Biotech, Beijing, China). For primers detecting the intraspecific intron content diversity of *C. fructicola*, the PCR condition was 4 min at 95 °C, then 20 cycles of 95 °C for 30 s, 60 °C for 30 s (decrease 0.5 °C per cycle), 72 °C for 2 min, then 30 cycles of 95 °C for 30 s, 50 °C for 30 s, 72 °C for 2 min, and then 7 min at 72 °C. The PCR condition for primers aimed at detecting *C. gloeosporioides* s.l. was the same as above except that the stage-II annealing temperature ranged between 65 °C and 55 °C and the stage-III annealing temperature was 55 °C. For PCR aimed at detecting *C. gloeosporioides* s.l., the reaction for each primer-isolate combination was conducted three times to assess repeatability. PCR products were analyzed with 1.2% agarose electrophoresis.

## Results

### Phylogenetic placement of the nine *C. gloeosporioides* s.l. isolates selected for mitogenome sequencing

**Fig. 1:**
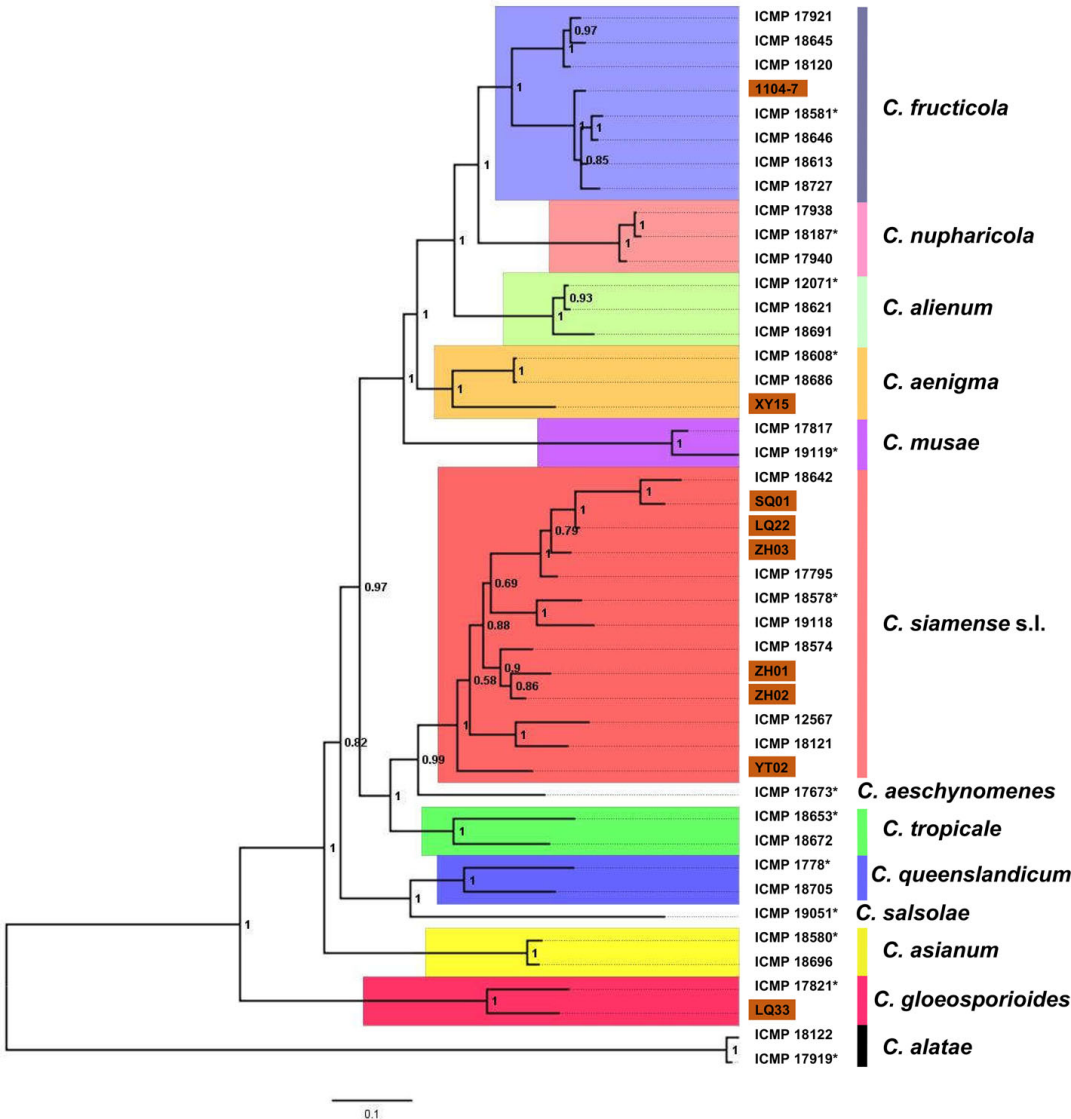
Phylogenetic placement of the *Colletotrichum gloeosporioides* s.l. isolates selected for mitogenome sequencing. The fifty percent majority rule consensus Bayesian tree was based on a 7-gene combined dataset (GADPH, CAL, SOD2, ACT, CHS, GS, TUB2). The alignment was partitioned, and the best DNA substitution model for each gene was determined by MrModelTest. *Colletotrichum alatae* isolates were used as the outgroup and the tree was mid-point rooted. Ex-type cultures are marked with ‘*’. For each node, posterior probability is displayed

The combined nuclear gene alignment dataset comprised 3,642 sites, among which 391 were parsimony-informative. The 50% majority rule consensus Bayesian tree (Fig. 1) was identical in topology to the one reported by Weir et al*.* [1]. Based on the dendrogram, we classified the nine isolates (shaded in brown) into four lineages, including *C. fructicola* (1104–7), *C. aenigma* (XY-15), *C. gloeosporioides* sensu stricto (LQ33, hereafter *C. gloeosporioides*), and *C. siamense* s.l. (SQ01, LQ22, ZH01, ZH02, ZH03, and YT02). *C. fructicola* has been reported to contain two clades indistinguishable in morphology and cultural appearance, both being widely distributed and associated with diverse hosts [1]. Isolate 1104–7 fell into the clade containing the ex-holotype ICMP 18581 (Fig. 1). The isolate XY-15 was classified as *C. aenigma*, a sister species of *C. fructicola*. Worldwide, *C. aenigma* has been known only from two collections, ICMP 18608 from *Persea americana* in Israel and ICMP 18686 from *Pyrus pyrifolia* in Japan (Fig. 1). On the phylogenetic tree, ICMP 18608 and ICMP 18686 were much closer to each other than to XY-15. Six isolates in this study (SQ01, LQ22, ZH01, ZH02, ZH03, and YT02) belonged to *C. siamense* s.l., a taxonomically controversial lineage being regarded as either a single species [1, 15] or a species complex [17]. As a species complex, ICMP 18642 and ICMP 19118 had been treated as independent species, being *C. hymenocallidis* [34] and *C. jasmini-sambac* [35] respectively. On the phylogenetic tree, the six *C. siamense* s.l. isolates located on three separate clades. SQ01, LQ22 and ZH03 were close to ICMP 18642 (*C. hymenocallidis*), ZH01 and ZH02 were close to ICMP 18574; YT02 was distal from all other *C. siamense* s.l. isolates, forming an independent branch with100% posterior probability, which supported the interpretation that YT02 represents a novel species if *C. siamense* s.l. is seen as a species complex. LQ33 was classified as *C. gloeosporioides* based on its close affinity to the epitype culture ICMP 17821 (Fig. 1).

### Introns and intergenic regions drive the significant size expansion of *C. gloeosporioides* s.l. mitogenomes

Prior to this study, five complete *Colletotrichum* mitogenomes were available in the GenBank database: three within the *C. acutatum* species complex (*C. acutatum*, *C. tamarilloi,* and *C. lupini*), one within the *C. orbiculare* complex (*C. lindemuthianum*), and one within the *C. graminicola* complex (*C. graminicola*). Through illumina DNA sequencing and assembly, we obtained the complete mitogenome sequences of the nine aforementioned *C. gloeosporioides* s.l. isolates. We compared these 14 mitogenomes in detail regarding their sizes, GC contents, gene contents, codon usage patterns and intron characteristics (Additional file 1: Table S3 to S7).

**Fig. 2:**
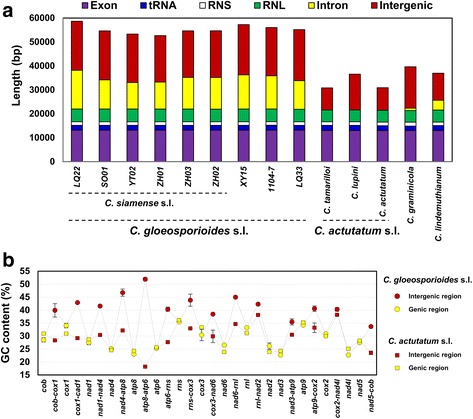
Size expansions and intergenic GC content elevations of the *Colletotrichum gloeosporioides* s.l. mitogenomes. **a** Size and content variations of *Colletotrichum* mitogenomes. Exon refers to oxidative phosphorylation protein-coding genes. RNS, small ribosomal RNA; RNL, large ribosomal RNA. **b** Differences in average GC contents between the genic and intergenic regions of *C. gloeosporioides* s.l. and *C. actutatum* s.l.. Plotted values are mean ± S.D., *n* = 9 for *C. gloeosporioides* s.l., *n* = 3 for *C. actutatum* s.l

The sizes of the nine *C. gloeosporioides* s.l. mitogenomes ranged between 52,671 and 58,666 bp (55,234 ± 1,863 bp), the sizes of the five mitogenomes outside of the complex ranged between 30,824 and 39,649 bp (34,975 ± 3,942 bp) (Additional file 1: Table S3, Fig. 2a); the average size of the former group was 1.58 times larger than the latter group. The average coding region lengths for mitogenomes within and outside of *C. gloeosporioides* s.l. were very close: 21,974 ± 46 and 21,509 ± 103 bp respectively. On the other hand, the corresponding values for the intergenic regions differed by 1.62 fold, being 20,228 ± 679 and 12,452 ± 3,551 bp respectively. *C. gloeosporioides* s.l. mitogenomes also contained significantly more introns. The three *C. acutatum* s.l. mitogenomes (*C. acutatum*, *C. lupini*, *C. tamarilloi*) contained no intron, *C. lindemuthianum* and *C. graminicola* contained three and one intron, with total length of 4,071 and 1,000 bp, respectively; the nine *C. gloeosporioides* s.l. mitogenomes, on the other hand, contained from six to 10 introns (Additional file 1: Table S4), with total length ranging from 11,057 to 16,199 bp.

When considering all 14 isolates, the summed length of intronic and intergenic regions closely predicted the total length of the mitogenome (*r* ^*2*^ = 0.999). When introns and intergenic regions were considered separately, the *r* ^*2*^ values were 0.954 and 0.900 respectively. Taken together, the *C. gloeosporioides* s.l. mitogenomes were much larger in size than those of any previously reported *Colletotrichum* groups, and the size expansion was attributable to length increases of both introns and intergenic regions, but not the coding regions.

### The *C. gloeosporioides* s.l. mitogenomes have significantly higher GC content compared with other *Colletotrichum* spp. due specifically to striking intergenic GC content elevation

The GC content of the nine *C. gloeosporioides* s.l. mitogenomes ranged between 33.8 and 34.6% (34.24 ± 0.21%), whereas the values for members not belonging to the complex ranged between 29.9 and 30.9% (30.34 ± 0.43%). The average value for the former group was significantly higher than the latter (t _[12]_ = 22.01, *p* < 0.001). In the genic regions (exons, introns, rRNAs), the average GC content values for members in and out of *C. gloeosporioides s.*l*.* were close: 31.07 ± 0.17% and 30.2 ± 0.05% respectively. In contrast, the corresponding values for the intergenic regions were 39.72 ± 0.25% and 30.76 ± 1.23% respectively, differing by almost 10%.

The *C. gloeosporioides* s.l. and *C. acutatum* s.l. mitogenomes were identical in gene order, which allowed us to compare their genic/intergenic GC content dynamics in more detail. Average GC content values for 17 genic and 14 intergenic regions were plotted (Fig. 2b). In all genic regions, the values of the two groups were very similar, but in all intergenic regions except *rnl-nad2* and *cox2-nad4l*, the values for *C. gloeosporioides* s.l. were much higher than for *C. acutatum* s.l. (Fig. 2b, Additional file 1: Table S5). Moreover, the GC content for all but one (*nad3*-*atp9*) *C. gloeosporioides* s.l. intergenic region was much higher than the neighboring genic regions, which however, was only observed with three out of 14 intergenic regions in *C. acutatum* s.l. (*nad4-atp8*, *rnl-nad2*, and *cox2-nad4l*).

### Comparative analysis of the sequence, codon usage, and gene organization features of the *C. gloeosporioides* s.l. mitogenomes

**Fig. 3:**
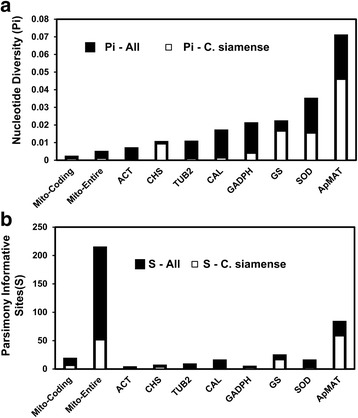
A comparison of the phylogenetic information conveyed by the mitogenome sequences and that of commonly used nuclear markers. **a** Nucleotide diversity value (π). **b** Parsimony informative sites (S). The calculations of π and s were either based on all *C. gloeosporioides* s.l. isolates (− All) or based on the *C. siamense* s.l. isolates alone (− C. siamense)

DNA sequences of the nine *C. gloeosporioides* s.l. mitogenomes were highly conserved. The mitogenome GBlocks alignment dataset contained 44,699 characters, among which merely 573 were polymorphic (12.8 per kb on average). Alignment of the protein-coding regions produced a dataset containing14,688 characters, among which merely 64 were polymorphic (4.36 per kb on average). The mitogenome nucleotide diversity value (π) was lower than all compared nuclear markers (Fig. 3a), indicating extremely slow overall evolution rate. Yet, due to length advantage, the mitogenome GBlocks alignment dataset contained the largest number of phylogenetically informative sites (212 in total, Fig. 3b), which was 2.6 times more than that of the most polymorphic nuclear marker, ApMAT (81 in total).

**Fig. 4:**
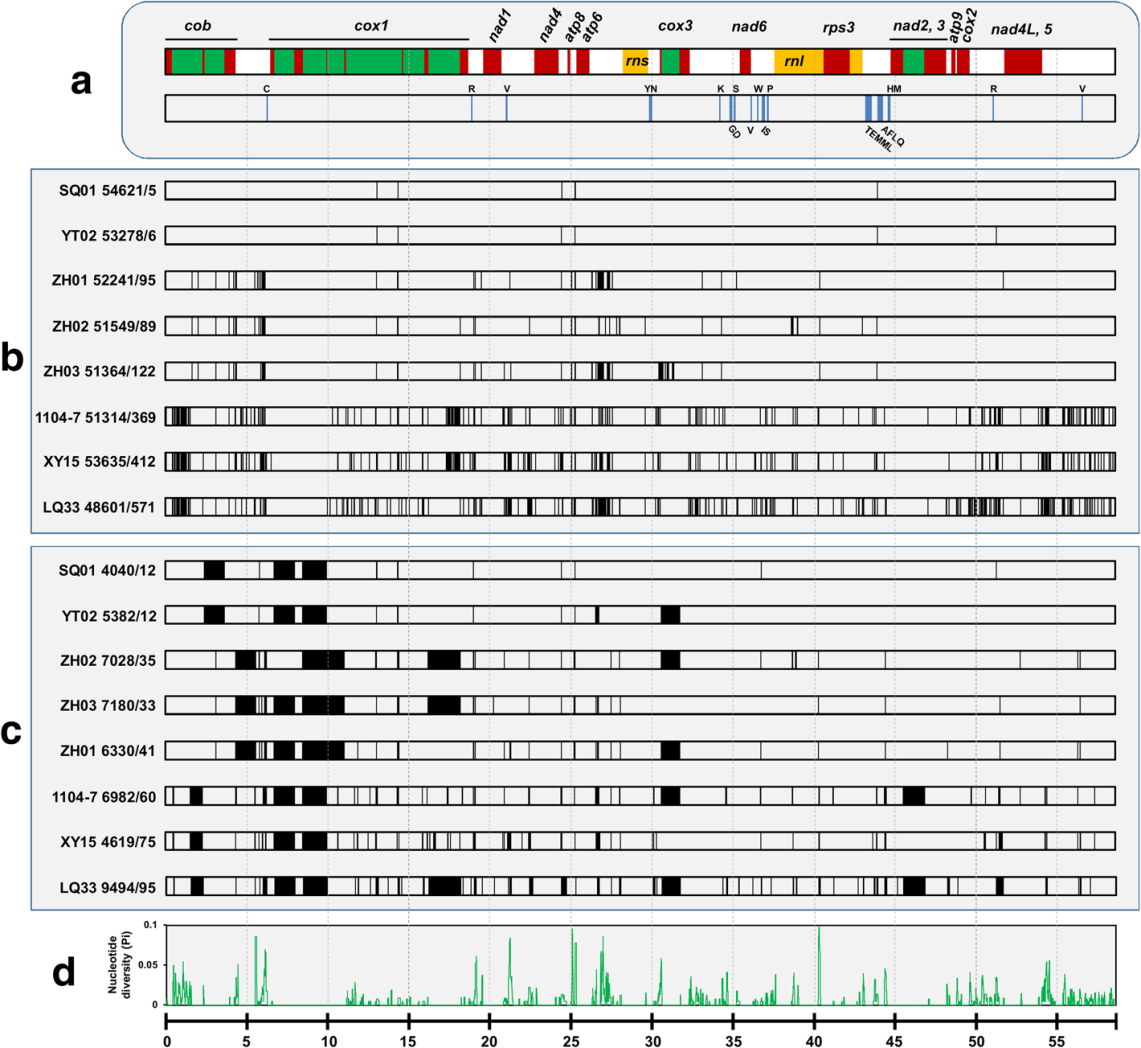
Sequence comparison of the *Colletotrichum gloeosporioides* s.l. mitogenomes. The features of LQ22 (*C. siamense* s.l.) and its comparison with other members are shown. **a** The LQ22 mitogenome gene features. Proteins (*exon in red, intron in green*) and rRNAs (*yellow*) are shown on top whereas tRNAs (*amino acid abbreviations*) are shown in bottom. **b** Distributions of LQ22 mismatch sites compared with indicated mitogenomes. Following isolate ID, total matched and mismatched sites (separated by dash line) are shown. **c** Distribution of LQ22 insertion sites compared with indicated mitogenomes. Following isolate ID, total insertion length and insertion sites are shown. **d** Sliding window analysis of nucleotide diversity (π) based on the nine mitogenomes (window length 50 bp, step size 5 bp, gaps omitted). Plots in all panels (a to d) share the same coordinate (in the bottom), the unit for which is kilo base (kb)

Each of the nine *C. gloeosporioides* s.l. mitogenomes encoded 44 identical genes transcribed in the same direction (Fig. 4a), including 14 oxidative phosphorylation proteins (three subunits of cytochrome oxidase [cox1-3], apocytochrome b [cob], three subunits of ATP synthetase [atp6, 8, 9], and seven subunits of NADH: ubiquinone oxidoreductase [nad1-6 and nad4L]), a ribosomal protein (rps3), large (rnl) and small (rns) rRNAs, and 27 tRNAs (Additional file 1: Table S4). The 27 tRNAs recognized all 20 standard amino acids, with three for valine, three for methionine, two for arginine, two for serine, and two for leucine. The order of the 44 genes was identical among all mitogenomes.

**Fig. 5:**
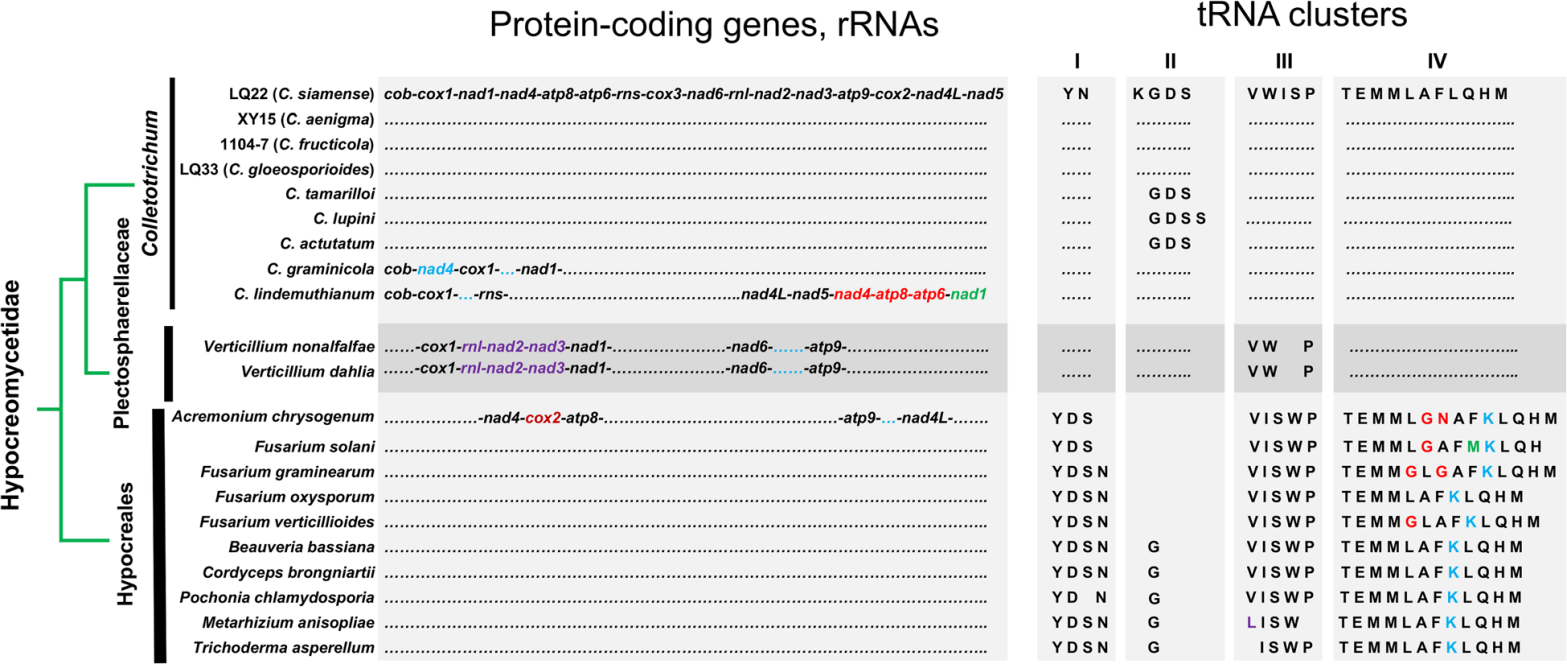
Mitogenome gene order evolution in the Hypocreomycetidae subclass. Dotted line indicates identical gene order compared with the reference (LQ22). Gene rearrangement events are highlighted in color. For tRNAs, the corresponding amino acid abbreviations are shown. GenBank accessions for *Colletotrichum* mitogenomes are listed in Additional file 1: Table S3. GenBank accessions for additional mitogenomes are as follows. *Verticillium nonalfalfae*, KR704425; *V. dahliae*, DQ351941; *Acremonium chrysogenum*, KF757229; *Fusarium solani*, NC_016680; *F. graminearum*, NC_009493; *F. oxysporum*, AY945289; *F. verticillioides*, NC_016687; *Beauveria bassiana*, EU371503; *Cordyceps brongniartii*, NC_011194; *Pochonia chlamydosporia*, KF479445; *Metarhizium anisopliae*, AY884128; *Trichoderma asperellum*, KR952346

Pairwise comparison identified several characteristics associated with the *C. gloeosporioides* s.l. mitogenomes: (1) mitogenomes within *C. siamense* s.l. were more similar to each other than to mitogenomes from different species lineages (Fig. 4b); (2) within *C. siamense* s.l., SQ01 and YT02 were nearly identical in DNA sequences (Additional file 2: Figure S1) despite falling far apart on the nuclear gene tree (Fig. 1); the mitogenomes of SQ01 and YT02 were identical except for eight InDel sites located either in introns or in intergenic regions, among which were six small-sized ones (one to five bp) and two large-sized ones (207 bp and 1,131 bp [*cox3* gene intron]); all six small-sized InDels were within single-nucleotide short DNA repeats; (3) nucleotide insertion/deletion events were less frequent compared with mutation events, and most large insertion/deletion events were due to intron gain/losses (Fig. 4c); (4) polymorphic sites distributed unevenly along the mitogenome, with the frequency being much higher in the intergenic and intronic regions than the coding regions (Fig. 4d); (5) a small coding region of the *cox3* gene showed strong signal of lineage-specific rapid divergence. Among the 64 polymorphic sites present in the mitogenome protein-coding regions, 36 were synonymous and 28 were nonsynonymous; these sites were distributed in eight of 15 genes (one in *nad2*, three in *rps3*, five in *nad4*, five in *nad5*, six in *cox1*, eight in *cox2*, nine in cob, and 27 in *cox3*). Strikingly, up to 18 of the 28 nonsynonymous sites were specifically located within a 142-bp long region of *cox3* (76 to 218 bp in the ORF), and most were singletons conferred by ZH03 (*C. siamense* s.l.) (Additional file 3: Figure S2). Further PCR and Sanger resequencing confirmed the nucleotide identity of ZH03 in this region (data not shown).

Codon usage frequencies were indistinguishable among the nine mitogenomes (Additional file 1: Table S6). All protein-encoding genes began with the typical AUG start codon except *cox3* with UUG, and all ended with the UAA stop codon except *cox3* and *nad6* with UAG. The most-frequently used codons were the same: UUA (L, 12.07–12.13%), AUA (I, 7–7.02%), UUU (F, 5.62–5.64%), and AAU (N, 4.29–4.33%). On the other hand, UGC (C), CGC (R), CGG (R), AGG (R) were universally absent. In addition, a number of codons were under-represented (relative frequency < 1%), e.g. CGA (R, 0.2%), CUC (L, 0.2%), ACG (T, 0.2–0.4%), AAG (K, 0.2–0.7%), CAG (Q, 0.4%), CCC (P, 0.4%), GGC (G, 0.4–0.7%), GUC (V, 0.7%), ACC (T, 0.7%), UGG (W, 0.7%), and CCG (P, 0.9%). Such codon usage patterns were similarly observed in other *Colletotrichum* mitogenomes (Additional file 1: Table S6). Among all *Colletotrichum* mitogenomes, the CGC and AGG codons coding for lysine were absent; moreover, codons present at higher frequency tended to favor A and T nucleotides, in accordance with reports in other fungi [22].

The nine *C. gloeosporioides* s.l. mitogenomes shared identical orders of protein-coding genes, rRNAs and tRNAs. We further examined the extent of such order conservation in the Hypocreomycetidae subclass. For protein-coding genes and rRNAs (Fig. 5, left), the *C. gloeosporioides* s.l. order was conserved among all *C. actutatum* s.l. members and all Hypocreales members except *Acremonium chrysogenum*, suggesting that this order represents the ancestral status of Hypocreomycetidae. The orders of *C. graminicola*, *C. lindemuthianum*, *Verticillium nonalfalfae*, *V. dahlia* and *A. chrysogenum* were slightly different, involving one or two DNA rearrangement events (Fig. 5, left).

In each of the nine *C. gloeosporioides* s.l. mitogenomes, 22 of 27 tRNAs clustered together and formed four groups, which were between *rns* and *cox3* (cluster I, *trnY*, *trnN*), between *cox3* and *nad6* (cluster II, *trnK*, *trnG*, *trnD*, *trnS*), between *nad6* and *rnl* (cluster III, *trnV*, *trnW*, *trnI*, *trnS*, *trnP*), and between *rnl* and *cox1* (cluster IV, *trnT*, *trnE*, *trnM*, *trnM*, *trnL*, *trnA*, *trnF*, *trnL*, *trnQ*, *trnH*, *trnM*). We identified and compared tRNAs belonging to these four clusters in different Hypocreomycetidae mitogenomes (Fig. 5, right). In general, the extent of tRNA content and organization variation correlated well with taxonomic distance. For instance, in cluster I, all *Verticillium* and *Colletotrichum* members had ‘Y N’ whereas Hypocreales members had ‘Y D S’ or ‘Y D S N’; in cluster II, all *Verticillium* and *Colletotrichum* members had at least three genes, ‘G D S’, whereas Hypocreales members only had a ‘G’ or none at all; and in cluster IV, all Hypocreales members had a *trnK* (‘K’) gene, which was absent in both *Verticillium* and *Colletotrichum*.

Within the genus *Colletotrichum*, tRNA organization of *C. graminicola* and *C. lindemuthianum* was identical to that of *C. gloeosporioides* s.l. members. The three species belonging to the *C. actutatum* complex, on the other hand, had different tRNA organization in cluster II. Compared with *C. gloeosporioides* s.l. (‘K G D S’), the tRNA organization for *C. tamarilloi* and *C. actutatum* was ‘G D S’ whereas that for *C. lupini* was ‘G D S S’. Sequence comparison indicated that the *trnS* duplication in *C. lupini* was due to a lineage-specific 5,708 bp DNA insertion event (data not shown).

### Significant intron content variation among the *C. gloeosporioides* s.l. mitogenomes

**Fig. 6:**
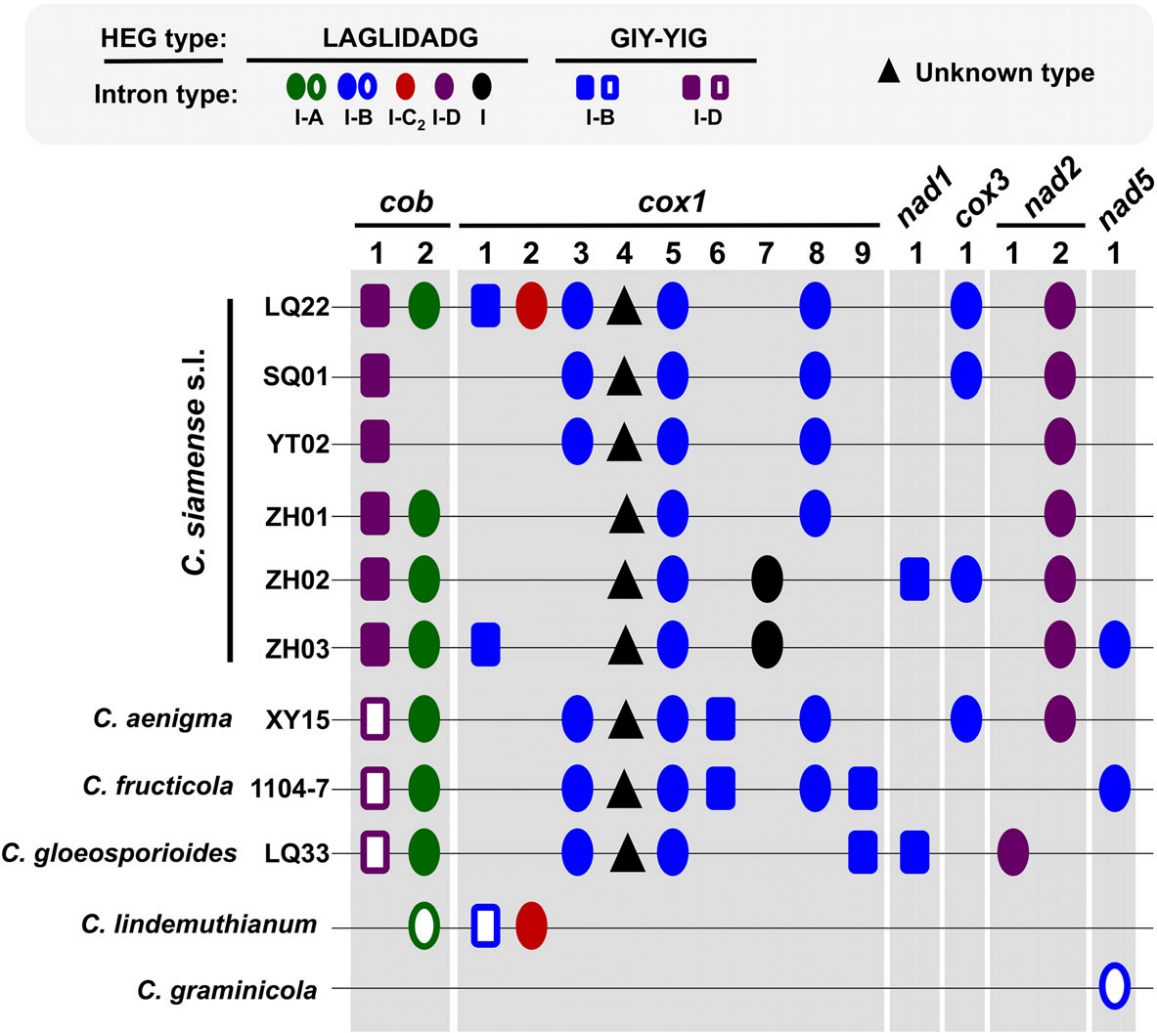
The *Colletotrichum gloeosporioides* s.l. mitogenome introns. The 71 type I introns (further classified into type I-A, I-B, I-C_2_, and I-D) locate at 16 conserved positions in six genes, and most harbor homing endonuclease genes (classified into ‘LAGLIDADG’ and ‘GIY-YIG’ types). Filled and open symbols mark introns with slightly different features. For *cob-1*, the intron sizes of XY15, 1104–7 and LQ33 are smaller. For *cox1-1* and *nad5-1*, the introns in *C. lindemuthianum* and *C. graminicola* have different intron phases. Please refer to Additional file 1: Table S7 for detailed information about the positions, codon phases and lengths of introns

In total, seventy-one introns were identified among the nine *C. gloeosporioides* s.l. mitogenomes (Fig. 6, Additional file 1: Table S7). These introns ranged between 997 and 3,516 bp in size, and were located at 16 conserved positions within six oxidative phosphorylation protein-encoding genes, which included nine in *cox1*, two in *cob,* two in *nad2*, one in *nad1*, one in *cox3*, and one in *nad5*. All identified introns belonged to the group I intron family except for cox1-4, which lacked distinguishable features for classification. The group I introns could be further subdivided into type I-A (cob-2, seven in total), type I-B (cox1-1, cox1-3, cox1-5, cox1-6, cox1-8, cox1-9, nad1-1, cox3-1, nad5-1; 35 in total), type I-C_2_ (cox1-2, one in total), and type I-D (cob-1, nad2-1, nad2-2; 17 in total). The cox1-7 intron, present in ZH02 and ZH03, belonged to type I but could not be further subdivided. Except for cox1-4, introns at all positions harbored homing endonuclease genes (HEGs) known to direct intron mobility. Intronic HEGs could be further divided into LAGLIDADG and GIY-YIG families. Forty-five introns at 10 positions harbored LAGLIDADGs whereas 17 introns at another five positions harbored GIY-YIGs.

In general, introns at the same position were highly similar in terms of length and sequence. However, the cob-1 site was an exception, where the intron sizes of XY15, 1104–7, and LQ33 (1,259 to 1,274 bp) were much smaller than the *C. siamense* s.l. members (LQ22, SQ01, YT02, ZH01, ZH02, ZH03, 1909 to 1912 bp) (Additional file 1: Table S7). Sequence comparison showed the size difference was due to non-homologous sequences at the 3′ region (data not shown).

High-level intron content variation was observed among the nine *C. gloeosporioides* s.l. mitogenomes. Each mitogenome contained a unique set of introns. Among the 16 intron positions, cob-1, cox1-4, and cox1-5 were the only three positions conserved among all genomes (Fig. 6). Considerable intron presence-absence polymorphism was also observed within *C. siamense* s.l., for which nine out of 12 positions were polymorphic. The presence-absence polymorphism was also observed between SQ01 and YT02, two isolates being almost identical in mitogenome sequence. These result demonstrated rapid evolutionary dynamics of introns in *C. gloeosporioides* s.l.

**Fig. 7:**
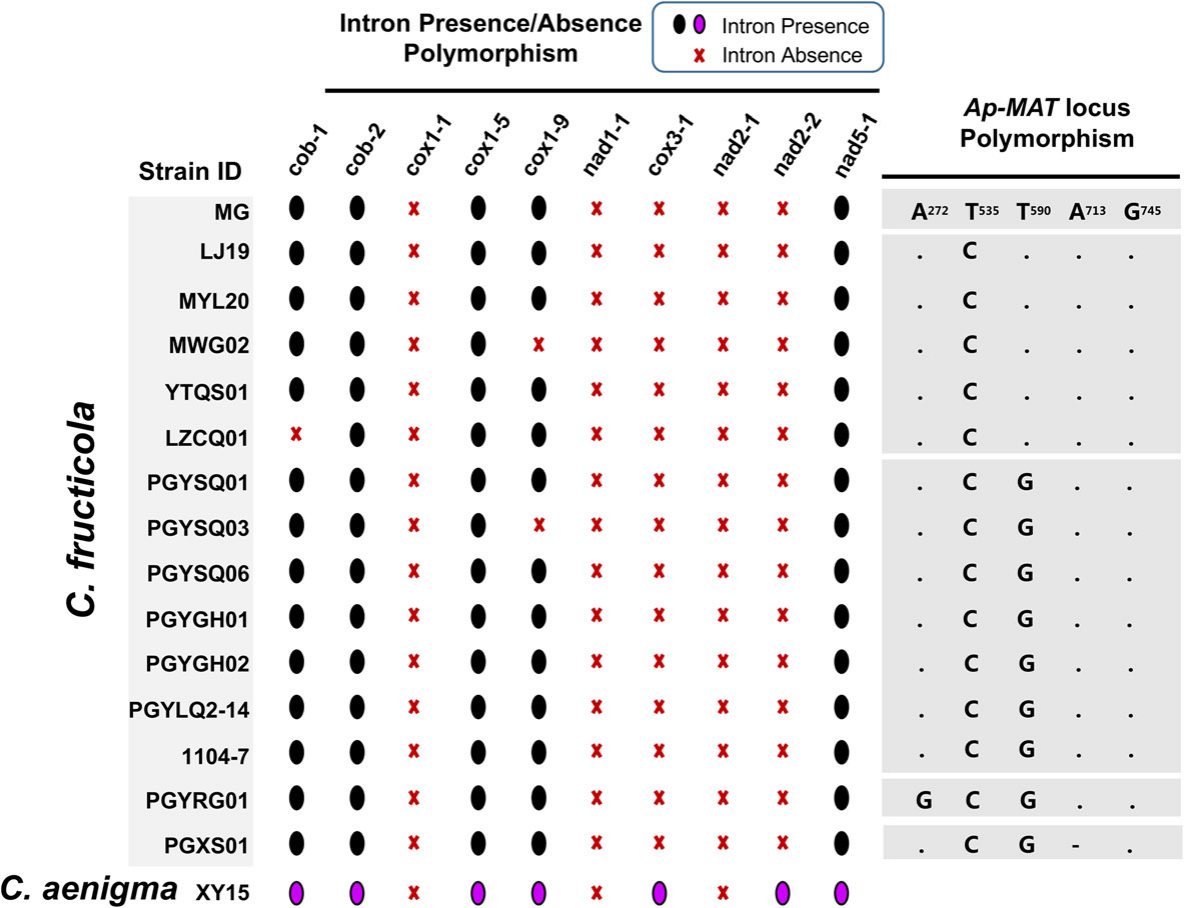
Intraspecific intron content polymorphism in *Colletotrichum fructicola*. The intron presence-absence patterns of 15 surveyed isolates, together with the nucleotides of these isolates at five polymorphic sites within a 762 bp long alignment of *ApMAT* locus DNAs are shown. The intron content of the *C. aenigma* isolate XY15 is shown as a comparison. GenBank accessions of the *ApMAT* locus sequences are listed in Additional file 1: Table S1

We further surveyed the intraspecific intron content variation in another *C. gloeosporioides* s.l. species, *C. fructicola*, for which we analyzed 10 intron positions and 15 isolates (including 1104–7) (Fig. 7). These *C. fructicola* isolates were isolated from different hosts and from different locations (Additional file 1: Table S1), and contained five *ApMAT* haplotypes, and were thus genetically different. The intron content patterns of these 15 isolates could be classified into three types. Eleven isolates, containing all five *ApMAT* haplotypes, had completely identical intron content. Three other isolates (MWG_02, PGYSQ03, LZCQ01) had slightly different intron contents. The ApMAT nucleotide diversity value calculated from the 15 *C. fructicola* isolates was 0.00126, much lower than that of *C. siamense* s.l. (based on LQ22, SQ01, YT02, ZH01, ZH02, ZH03, π = 0.0458). Thus, both intron content and *ApMAT* locus sequence data supported a lower level of intraspecific genetic diversity of *C. fructicola*.

The unique conservation of cob-1, cox1-4, and cox1-5 introns in *C. gloeosporioides* s.l. isolates indicated their potential value for molecular identification of taxa within *C. gloeosporioides* s.l.. We designed four primer pairs targeting these introns (primer design strategy shown in Additional file 4: Figure S3). The primer pair P_1_/P_2_, P_7_/P_8_ targeted cob-1 and cox1-5 respectively; the four primer binding sites were in exons and were highly conserved among at least four divergent *Colletotrichum* species complexes (*C. gloeosporioides* s.l., *C. actutatum* s.l., *C. orbiculare* s.l., *C. graminicola* s.l.; Additional file 4: Figure S3b). The primer pairs P_3_/P_4_ and P_5_/P_6_ recognized two separate regions inside cox1-4, supposed to be unique to *C. gloeosporioides* s.l.. The designed primer pairs were tested against eight *C. gloeosporioides* s.l. isolates and five *C. actutatum* s.l. isolates (results shown in Additional file 5: Figure S4). All primer pairs produced strong and specific bands when using the *C. gloeosporioides* s.l. DNAs as templates, indicating high amplification efficiencies. When using the *C. actutatum* s.l. DNAs as templates, P_3_/P_4_ and P_5_/P_6_ generated amplifications from time to time, the size of which matched that expected for *C. gloeosporioides* s.l., indicating poor primer specificity. P_7_/P_8_ produced ~1,440 bp amplicons for all *C. gloeosporioides* s.l. isolates and ~120 bp amplicons for all *C. actutatum* s.l. isolates, the ~120 bp amplicons were relatively weak and indistinctive from primer dimers, so this can be problematic for identification purposes. For P_1_/P_2_, all *C. actutatum* s.l. isolates produced an expected ~ 360 bp band whereas all *C. gloeosporioides* s.l. isolates produced bands larger than 1,500 bp (~1650 bp for XY15, 1104–7, LQ33; and ~ 2300 bp for other isolates), amplicon size differences among the *C. gloeosporioides* s.l. isolates perfectly matched their cob-1 intron sizes as reported above. In repeated experiments, P_1_/P_2_ always produced strong and specific bands for both *C. actutatum* s.l. and *C. gloeosporioides* s.l., providing clear distinction between these groups.

### Implications of mitogenome-based phylogeny

We first constructed maximum likelihood (ML) phylogenetic trees based on either mitogenome proteins or mitogenome protein-coding DNAs. On both trees, all nine *C. gloeosporioides* s.l. isolates clustered into a well-support monophyletic clade neighbored by *C. lindemuthianum*, *C. actutatum* s.l. and *C. graminicola* (Additional file 6: Figure S5). These species together represented the *Colletotrichum* genus. On the protein tree, the genus *Colletotrichum* was neighbored by *Verticillium* spp.; the two genera together were further neighbored by Hypocreales. Such a topological pattern was in accordance with the genetic relationships of Glomerellales, Plectosphaerellaceae and Hypocreales [36, 37], and supported the previous phylogenetic placement of *C. gloeosporioides* s.l. based on nuclear markers. No phylogenetic structure was detected within *C. gloeosporioides* s.l. members on either tree, due to an exceptionally low level of sequence variations.

**Fig. 8:**
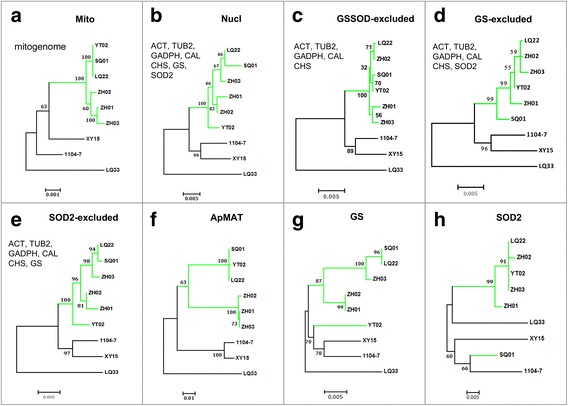
Maximum likelihood phylogenetic trees constructed from different datasets. For concatenation tree, markers used for concatenation are shown. Best nucleotide substitution models for ‘Mito’, ‘Nucl’, ‘GSSOD-excluded’, ‘GS-excluded’, ‘SOD2-excluded’, ‘ApMAT’, ‘GS’, and ‘SOD’ phylogenetic trees were determined by MrModelTest, which were ‘GTR + G + I’, ‘HKY + G + I’, ‘HKY + I’, ‘HKY + I’, ‘HKY + G + I’, ‘HKY + I’, ‘HKY’, and ‘HKY + G’ respectively. Numbers at the nodes are bootstrap values based on 1,000 replicates. Green color indicates *C. siamense* s.l

A ML phylogenetic tree was further constructed based on the 44,699 bp long mitogenome Gblocks alignment (Fig. 8a). The nine isolates formed four well-separated clades, corresponding to *C. siamense* s.l. (green color), *C. aenigma* (XY15), *C. fructicola* (1104–7), and *C. gleosporioides* (LQ33) respectively. Such topological pattern correlates well with their distributions on the nuclear marker-based phylogenetic tree (Figs. 1 and 8b). Within *C. siamense* s.l., however, a strong discordance was observed between the mitogenome and nuclear trees. The ‘Mito’ tree contained two distinctive clades, one clade included ZH01, ZH02, and ZH03 whereas the other clade included SQ01, YT02, and LQ22 (Fig. 8a), SQ01, YT02, and LQ22 were almost indistinguishable. On the nuclear tree (Fig. 8b), the six *C. siamense* s.l. isolates formed two groups with YT02 being separated from all other isolates. The striking different phylogenetic positions of YT02 highlighted a discordance between mitogenome and nuclear markers in *C. siamense* s.l.. Preliminary analysis showed that the phylogenetic discordance could be eliminated by excluding the GS and SOD2 markers from analysis (Fig. 8 c, d, g, h). The *ApMAT* marker-based phylogeny (Fig. 8f) was highly similar in topology compared with that based on mitogenome.

Shimodaira-Hasegawa (SH) test was further performed to test marker incongruence (Table 1). The mitogenome-based tree (‘Mito’ tree, Fig. 8a) was compared with the tree based on concatenated nuclear loci (‘Nucl’ tree, Fig. 8b). As a control, the ‘Mito’ tree was also compared with the ‘GSSOD-excluded’ tree (Fig. 8c), the ‘GS-excluded’ tree (Fig. 8d), the ‘SOD-excluded’ tree (Fig. 8e), and the ‘ApMAT’ tree (Fig. 8f). SH tests were carried out against different nuclear marker datasets. As shown in Table 1, at 0.01 significance level, all nuclear markers equally supported the tested trees when comparing the ‘Mito’ tree with either the ‘ApMAT’ tree or with the ‘GSSOD-excluded’ tree. When comparing the ‘Mito’ tree with the ‘Nucl’ tree, ACT, TUB2, CAL, GADPH, CHS and SOD2 equally supported the two trees whereas GS strongly favored the ‘Nucl’ tree (*P* = 0, ‘Diff -ln L’ = −145.73). Such strong topological bias of the GS marker, however, was not observed when comparing the ‘Mito’ tree with the ‘GS-excluded’ tree (GS marker excluded for concatenation). In actual fact, the GS marker favored the ‘Mito’ tree slightly more than the ‘GS-excluded’ tree (*P* = 0.0201, ‘Diff -ln L’ = 17.18). Thus, the topological incongruence between the ‘Mito’ tree and the ‘Nucl’ tree was mainly caused by GS. The SOD2 marker strongly favored the ‘GS-excluded’ tree over the ‘Mito’ tree (*P* = 0.0076, ‘Diff -ln L’ = −39.27), while equally supporting the ‘Mito’ tree and the ‘Nucl’ tree (*P* = 0.203, ‘Diff -ln L’ = −2.01), indicating that the effect of GS masked that of SOD2.

**Table 1:** The Shimodaira-Hasegawa (SH) test result

Tree^a^	Value﻿ types^b^	ACT	TUB2	CAL	GADPH	CHS	GS	SOD2
Mito	-ln L	435.06	1154.96	1272.82	512.87	504.86	1754.51	815.79
ApMAT	Diff -ln L	0	−18.64	−4.96	0	3.18	−6.38	−2.01
	*P*-value	/	0.0433	0.1901	/	0.2279	0.1519	0.203
Nucl	Diff -ln L	0	−10.63	−14.26	13.98	12.48	−145.73	−2.01
	*P*-value	/	0.1872	0.0893	0.0798	0.0819	0*	0.203
GSSOD-excluded	Diff -ln L	0	−18.64	−4.75	−5.24	−2.77	17.18	−2.01
	*P*-value	/	0.0447	0.204	0.1472	0.276	0.0203	0.203
GS-excluded	Diff -ln L	0	−10.63	−4.75	13.98	−20.16	17.18	−39.27
	*P*-value	/	0.1926	0.204	0.0756	0.0626	0.0201	0.0076*
SOD-excluded	Diff -ln L	0	−10.63	−14.26	13.98	12.48	−145.73	−2.01
	*P*-value	/	0.1894	0.0905	0.0776	0.0823	0*	0.203

^a^ The ‘Mito’ tree was compared with the ‘ApMAT’ tree, concatenated nuclear tree (‘Nucl’), ‘GSSOD-excluded’ tree, ‘GS-excluded’ tree, and ‘SOD-excluded’ tree based on indicated datasets. Tree topologies are shown in Fig. 8

^b^For the ‘Mito’ tree, ‘-ln L’ represents the -ln^(likelihood score)^ value. For each compared tree, ‘Diff -ln L’ represent the differences between their -ln^(likelihood score)^ values and that of the ‘Mito’ tree. Negative value indicates that the dataset supports the tested tree better than the ‘Mito’ tree. The *P*-value for each test is reported and ‘*’ indicates *P* < 0.01. ‘/’ indicates equal -ln^(likelihood score)^ values in which case SH test could not be performed

Given the significant biased effects of GS and SOD2, we performed further concatenation phylogenic analysis of the 42 *C. gloeosporioides* s.l. isolates by excluding either the GS marker alone (Additional file 7: Figure S6a), or the GS marker as well as the SOD2 marker (Additional file 7: Figure S6b) from the alignment dataset. When only GS was excluded, all species lineages still formed monophyletic clades and the tree topology was in general identical to the previous seven-locus tree (Fig. 1). The six *C. siamense* s.l. isolates formed two clades, and clade one includes all isolates except SQ01. When both GS and SOD2 were excluded, all *C. siamense* s.l. isolates clustered into one well-supported clade (posterior probability = 1), and no further genetic structure could be recognized; all other species lineages could still be recognized with high confidence except for *C. fructicola*, for which the two intraspecific groups formed sister clades. In general, it was clear that presence/absence of the GS and SOD2 markers strongly affected the phylogenetic positions of YT02 and SQ01; thus, lineage-specific divergences of GS and SOD2 were the main sources causing phylogenetic incongruence.

We further examined polymorphism sites in the GS and SOD2 alignments for sequence patterns potentially explaining their lineage-specific divergences (Additional file 8: Figure S7). The GS marker is made up of a large partial intron (~850 bp) and a very small coding region (8 bp). The alignment dataset contained 31 polymorphic sites for the *C. siamense* s.l. isolates, among which 14 were YT02-specific mutations; these 14 mutations occurred throughout the alignment, and 12 were transitions, including eight C to T (or G to A) and four T to C (or A to G). The SOD2 marker located in the coding region (374 bp in *C. siamense* s.l.), and the alignment contained 16 polymorphic sites among which 12 were SQ01-specific mutations, also dispersed in distribution. All SQ01-specific mutations were synonymous and located at the third codon positions except 216^A to G^ and 217^G to A^, which together caused a ‘AGC’ (Ser) to ‘GAC’ (Asp) codon change. In general, we observed no obvious selection-indicative sequence pattern with either the GS or SOD2 marker, in accordance with a hypothesis that incomplete lineage sorting has driven the observed divergences.

## Discussion

The fungal species complex *C. gloeosporioides* s.l. is economically and ecologically important, yet taxonomically challenging. In this study, mitogenomes representing nine taxonomic lineages were first determined, which provides useful resources for further genetic studies. Many features observed with the nine mitogenomes are also typical of the Hypocreomycetidae subclass and even the Sordariomycetes class; for instance, the transcription of all protein-encoding genes in the same direction, the presence of *rps3* gene within the ribosomal large subunit, the conserved ordering of protein-encoding genes, tRNAs, and rRNAs, and the prevalence of HEG-containing introns. Compared with known *Colletotrichum* mitogenomes, however, the *C. gloeosporioides* s.l*.* mitogenomes are also distinctive for containing longer intergenic regions with elevated GC content and for being much richer in introns.

GC bias is common with both nuclear and mitochondrial genomes [38], and the GC contents of closely-related lineages can differ significantly [39]. The average intergenic GC content of *C. gloeosporioides* s.l. mitogenomes is around 10% higher than that of the genic regions. Although it remains to be determined whether such striking difference is prevalent in filamentous fungi, the lack of elevation in other *Colletotrichum* mitogenomes indicates a lineage specificity. The GC content elevation is prevalent among most intergenic regions, indicating that the elevation is not due to regional horizontal DNA transfer. Both neutral evolutionary events, such as biased mutation or biased gene conversion, and natural selection have been implicated to drive GC content evolution [40]. The striking GC content elevation observed in this study is specific to the intergenic regions where DNAs are under the least functional constraint, which would indicate neutral evolutionary forces. Mitochondrial DNA mutations, however, are AT biased due to increased likelihood of cytosine deamination or guanine oxidation [38]. Thus, if the GC content elevation is evolutionarily neutral, biased gene conversion would be a more likely mechanism. Gene conversion, however, relies on DNA mismatch repair system and DNA recombination events, the occurrence and frequency of which in *C. gloeosporioides* s.l. must be confirmed.

Regarding the natural selection hypothesis, genome GC content elevation has been implicated to confer an adaptation advantage toward high temperature, UV exposure or conditions where high DNA stability is required [41, 42]. If the intergenic DNAs are rich with regulatory elements, the intergenic GC content elevation observed here could in principle be relevant to the modulation of mitochondrial gene expressions, a mechanism proposed to explain the correlation between GC content and stress tolerance observed with monocot plants [43].

While the *C. gloeosporioides* s.l. mitogenome sequences are highly conserved, particularly at the coding regions, we have identified a 142-bp-long region in the *cox3* ORF showing strong lineage-specific divergence. The short region contains 23 polymorphic sites, the frequency of which is over 300-fold higher than the rest of the coding regions. Moreover, up to 18 sites are non-synonymous, indicating functional relevance of the nucleotide changes. Most polymorphic sites are ZH03-specific singletons, demonstrating that this DNA region evolves differently in ZH03, likely involving recombination or horizontal transfer events. While its evolutionary origin and biological implications are unclear, the presence of such feature emphasizes the advantage of phylogenetic analysis based on entire mitogenome over partial mtDNA sequence.

Broad intron content variation exists among fungal mitogenomes, where both interspecific and intraspecific variations have been documented. Moreover, intron content variation has been a main source explaining the size variation commonly observed with closely-related mitogenomes [22, 44–47]. In this study, significant intron content variation exists both among and within *C. gloeosporioides* s.l. species, and most introns contain HEGs of either LAGLIDADG or GIY-YIG type. Such pattern suggests that horizontal intron cycling [48], a mechanism known to regulate intron gain and loss process, also plays a significant role in shaping the intron dynamics of *C. gloeosporioides* s.l.

*Colletotrichum gloeosporioides* was established in the 1950s, but understanding of its taxonomic position has shifted over time. The current broad concept (*C. gloeosporioides* s.l.) is defined by ITS phylogeny [1], and morphological phenotypes alone are unreliable for its identification from other lineages, such as *C. boninense* species complex [49] and *C. cliviae* [34]. Due to historical reason, many GenBank-deposited ‘*C. gloeosporioides*’ sequences are inaccurately annotated [6], which can be a problem for ITS-based identification. The development of a secondary *C. gloeosporioides* s.l. identification marker is thus advantageous. Introns at three positions (cob-1, cox1-4, and cox1-5) are conserved among all *C. gloeosporioides* s.l. mitogenomes; however, none of them was observed in mitogenomes outside of the *C. gloeosporioides* s.l. complex, indicating a potential utility for PCR-based detection. We further tested four primer pairs and identified P_1_/P_2_ targeting cob-1 as the one with the highest specificity. The P_1_/P_2_ primer pair identifies *C. gloeosporioides* s.l. based on amplicon size increase, instead of amplicon presence; such a strategy is more advantageous because: (1) all surveyed samples should produce amplification bands indicating successful PCR reaction, thus this is a good positive internal control of DNA template and PCR reagents; and (2) the strategy dramatically reduces the likelihood of non-specific primer binding and non-specific amplification, thus improving detection specificity and repeatability, which is a critical advantage in PCR-based detections. The P_1_/P_2_ primer-binding sites are highly conserved among very divergent species complexes (*C. gloeosporioides* s.l., *C. actutatum* s.l., *C. orbiculare* s.l., and *C. graminicola* s.l.), and are thus likely to be applicable across the whole *Colletotrichum* genus. Another advantage of the P_1_/P_2_ primer pair is that it detects mtDNA, which has a high copy number and is easily amplified. However, the P_1_/P_2_ effectiveness has been validated with only relatively few *C. gloeosporioides* s.l. and *C. actutatum* s.l. isolates, so it is critical to test its practical efficiency and specificity with more representative isolates in the future.

For recently divergent lineages, gene tree/species tree discordance is a critical factor confounding species inference. One approach to overcome this bias is to improve the number, representativeness, and independence of analyzed genetic markers. Taxonomic studies in *C. gloeosporioides* s.l. have relied almost entirely on nuclear markers, whereas we have diverged from this pattern by exploring the merit of a mitogenome tree. The length advantage of mitogenome alignment overcomes its drawback of low-level nucleotide diversity. In the present study, the final mitogenome alignment contains 2.6 times more phylogenetically informative sites than that of ApMAT*.* The alignment dataset produces a very solid tree in high resolution, based on which the relationships of very nascent lineages can be inferred. The merit of complete mitogenome sequences, however, lies not only in the generation of a gene tree in high resolution, but perhaps more importantly in the fact that it provides information about the lineage evolutionary histories based on a factor different from all prevailing markers in use. By comparing these independent markers, one can have a better chance of teasing out phylogenetic signals not related to species divergence, and delimitating nascent species with increased accuracy. The relationship between YT02 and SQ01 analyzed in this study is a striking illustration. Neither phylogeny based on ApMAT nor phylogeny based on the concatenation of ACT, TUB2, GADPH, CAL, CHS markers separate the two isolates. However, if GS and SOD2 markers were further included in the concatenation, the resulting tree would separate the two isolates into different *C. siamense* s.l. species. Without additional information, one would hesitate in deciding which phylogeny to put trust on. Yet, based on the information that SQ01 and YT02 have almost identical mitogenomes and the information that the mitogenome phylogeny is congruent with all other markers except GS and SOD2, one can confidently postulate that the strong lineage-specific sequence divergences of GS and SOD2 are due to evolutionary events independent of species divergence. Further examination of polymorphism sites did not identify evidence of selection, thereby indicating incomplete lineage sorting, one of the most common reasons causing species tree/gene tree incongruence in nascent species, as a plausible reason. Our study shows that by integrating mitogenome sequence information, we have a better chance of discovering and correcting phylogenetic incongruity among the prevailing nuclear markers, which is a very critical step for the accurate recognition of nascent species. Compared with the mitochondrial genome, the nuclear genome is much larger and contains far more genes. Phylogenomic approaches, particularly ones considering the genome-wide variation of gene tree topologies, will allow one to delimit species boundaries to a much higher degree of confidence and accuracy. Such analysis is within expectation as the cost of whole-genome sequencing keeps dropping.

When applying mtDNA markers for phylogenetic studies, it is important to keep in mind that mtDNA is a cytoplasmic factor, the inherence and population dynamics of which can be quite different from nuclear DNA. The genetic exchange of mtDNA is achieved via either vegetative hyphal cell fusion limited by vegetative compatibility (VC), or via maternal (perithecial parent) inheritance limited by female fertility. The exchange of nuclear DNA, on the other hand, occurs in a biparental direction and is limited by cross fertility. When outcrossing is common, mutations of both nuclear and mtDNA can spread in the population. When outcrossing is rare, the spread of nuclear DNA mutations is restricted, generating genetic isolation. The spread of mtDNA mutations, on the other hand, may still be unaffected if vegetative compatibility is not affected. In *C. gloeosporioides* s.l., the cross-fertility phenotype is highly variable among isolates and species, whereas the presence and number of VC groups in most species are unknown. Nuclear and mtDNA may also spread among closely-related species through interspecific hybridization or hyphal fusion events. The combined interaction effects of potential intraspecific genetic isolation and interspecific genetic exchange of nuclear as well as cytoplasmic markers are hard to predict, so it is difficult to conclude whether nuclear or mitochondrial markers are more advantageous for species inference in this context. Similarly, phylogenetic trees from the two sources may not necessarily be fully congruent, and both may deviate away from true species relationships, which means that one must stay cautious about relying fully on either one for species inference. On the other hand, conclusions derived from concordant phylogenetic information derived from two sources will be more reliable than from a single source. For nuclear as well as mitochondrial DNA markers, it is also important that an adequate number of isolates is sampled so as that the observed variations approximate species-level changes more closely.

## Conclusion

The *C. gloeosporioides* s.l. mitogenomes characterized in this study are highly conserved in DNA sequence, gene content, gene transcribing direction, and gene order. Many mitogenome features are also conserved at higher taxonomic levels. On the other hand, these mitogenomes collectively have higher intron content and non-homogenous GC content distribution which were not observed with any previously reported *Colletotrichum* mitogenomes. Despite high sequence similarity, considerable intron content polymorphism exists among *C. gloeosporioides* s.l. lineages, indicating its potential application as a genetic diversity marker. Phylogenetic analysis demonstrates the usefulness of mitogenome in complementing nuclear genes for *C. gloeosporioides* s.l. species delimitation. Taken together, the *C. gloeosporioides* s.l. mitogenomes revealed in this study are important resources for further studies into the biology, genetics, and evolution of this economically important fungal species complex.

## Additional files

Additional file 1: Table S1. A list of isolated fungal strains with details about taxonomy, host, locality, and GenBank accessions of DNA markers. **Table S2**. A list of PCR primers. **Table S3**. General features of the *Colletotrichum* spp. mitogenomes. **Table S4**. Annotations of the mitogenome core genes (oxidative phosphorylation proteins, rRNAs, tRNAs). **Table S5.** Genic and intergenic regions GC contents (%) of *C. gloeosporioides* s.l. and *C. actutatum* s.l. mitogenomes. **Table S6**. Mitogenome codon usage frequencies. **Table S7**. A summary of the mitogenome intron features. (XLSX 55 kb)
Additional file 2: Figure S1. SQ01 (*C. siamense* s.l.) mitogenome gene features and its sequence comparison with that of YT02 (*C. siamense* s.l.). (PDF 362 kb)
Additional file 3: Figure S2. DNA sequence alignment of *C. gloeosporioides* s.l. mitogenomes at the short *cox3* gene coding region (76–218 bp) where ZH03 (*C. siamense* s.l.) showed strong lineage-specific sequence divergence. (PDF 847 kb)
Additional file 4: Figure S3. Primer design strategies for PCR-based identification of *C. gloeosporioides* s.l.. (PDF 394 kb)
Additional file 5: Figure S4. PCR amplification results of primers tested for differentiating *C. gloeosporioides* s.l. from *C. actutatum* s.l.. (PDF 1260 kb)
Additional file 6: Figure S5. Maximum likelihood phylogenetic trees based on concatenated mitochondrial proteins and concatenated coding DNAs. (PDF 322 kb)
Additional file 7: Figure S6. Fifty percent majority rule consensus Bayesian trees based on different gene concatenation schemes. (PDF 475 kb)
Additional file 8: Figure S7. DNA polymorphism sites in the GS (glutamine synthetase) and SOD2 (manganese-superoxide dismutase) alignments of *C. siamense* s.l. isolates. (PDF 464 kb)

## Abbreviations

ACT: Actin
BI: Bayesian inference
CAL: Calmodulin
CHS: Chitin synthase
CTAB: Cetyl trimethylammonium bromide
GAPDH: Glyceraldehyde-3-phosphate dehydrogenase
GS: Glutamine synthetase
HEGs: Homing endonuclease genes
ML: Maximum likelihood
mtDNA: Mitochondrial DNA
SH test: Shimodaira-Hasegawa test
SOD2: Manganese-superoxide dismutase
TUB2: β-tubulin 2
