# Supplementary material for: Comparative analysis of the mitochondrial genomes of Colletotrichum gloeosporioides sensu lato: insights into the evolution of a fungal species complex interacting with diverse plants
Source: BMC Genomics. 2017 Feb 15;18:171. doi: 10.1186/s12864-016-3480-x (PMC5311727; doi:10.1186/s12864-016-3480-x)
Supplement: Additional file 2: Figure S1. — SQ01 (C. siamense s.l.) mitogenome gene features and its sequence comparison with that of YT02 (C. siamense s.l.). (PDF 362 kb) [file 12864_2016_3480_MOESM2_ESM.pdf]

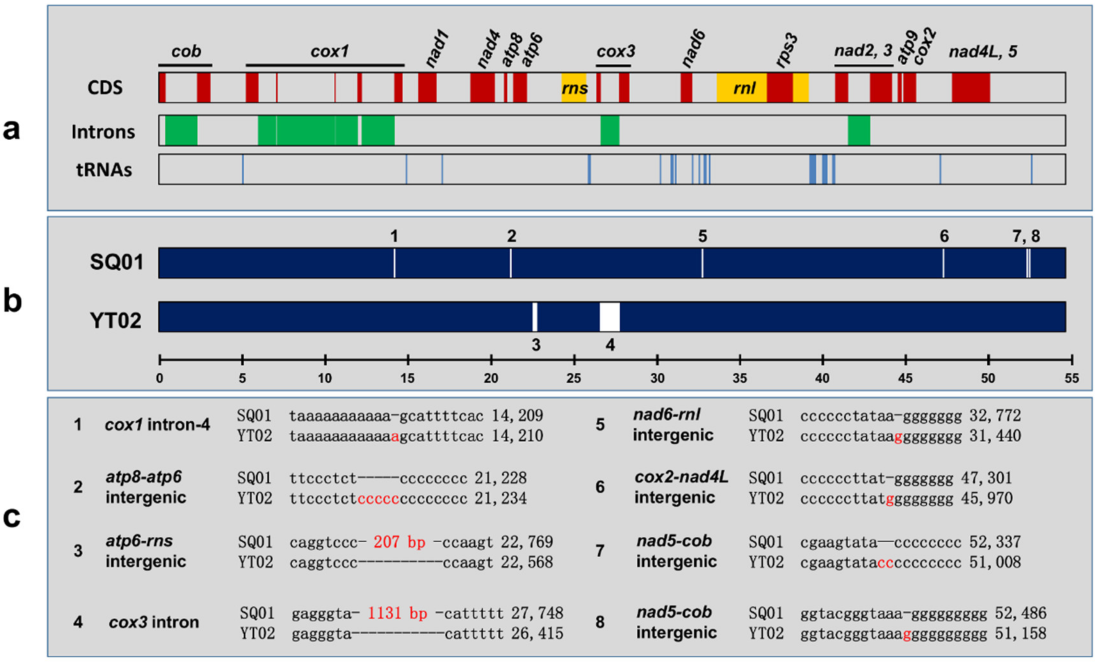

Figure S1. SQ01 (*C. siamense* s.l.) mitogenome gene features and its sequence comparison with that of YT02 (*C. siamense* s.l.). **a**. Schematic representation of the SQ01 mitogenome features. **b**. Positions of the eight InDel variable sites within SQ01 and YT02 mitogenomes. **c**. Local DNA sequence alignments at the InDel sites.
