# Supplementary material for: Comparative analysis of the mitochondrial genomes of Colletotrichum gloeosporioides sensu lato: insights into the evolution of a fungal species complex interacting with diverse plants
Source: BMC Genomics. 2017 Feb 15;18:171. doi: 10.1186/s12864-016-3480-x (PMC5311727; doi:10.1186/s12864-016-3480-x)
Supplement: Additional file 3: Figure S2. — DNA sequence alignment of C. gloeosporioides s.l. mitogenomes at the short cox3 gene coding region (76–218 bp) where ZH03 (C. siamense s.l.) showed strong lineage-specific sequence divergence. (PDF 847 kb) [file 12864_2016_3480_MOESM3_ESM.pdf]

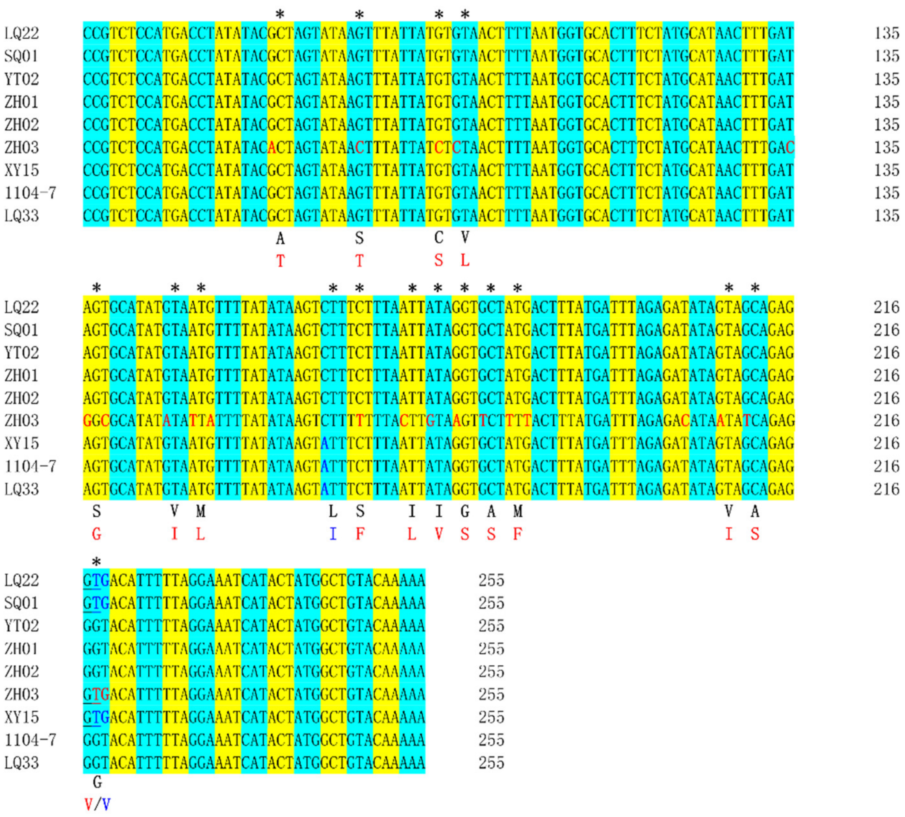

Figure S2. DNA sequence alignment of *C. gloeosporioides* s.l. mitogenomes at the short *cox3* gene coding region (76-218 bp) where ZH03 (*C. siamense* s.l.) showed strong lineage-specific sequence divergence. In the alignment, amino acid codons are shaded in cyan or yellow. SNP nucleotides in ZH03 are highlighted in red, whereas ones in other isolates are highlighted in blue. '*' marks nonsynonymous codon and the corresponding amino acid changes are shown in the bottom.
