# Supplementary figures and images for: Comparative analysis of the mitochondrial genomes of Colletotrichum gloeosporioides sensu lato: insights into the evolution of a fungal species complex interacting with diverse plants
Source: BMC Genomics. 2017 Feb 15;18:171. doi: 10.1186/s12864-016-3480-x (PMC5311727; doi:10.1186/s12864-016-3480-x)

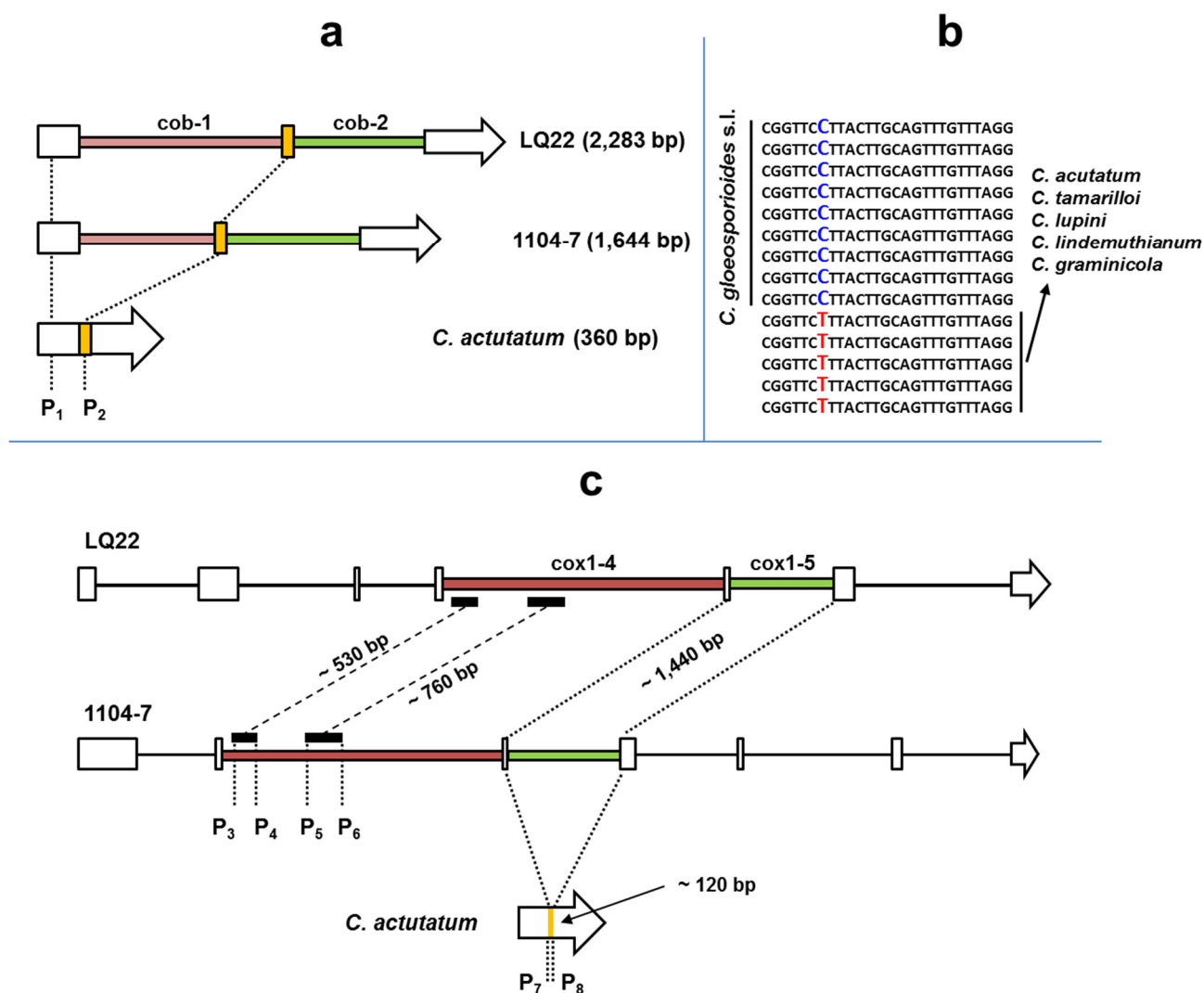

Supplement: Additional file 4: Figure S3. — Primer design strategies for PCR-based identification of C. gloeosporioides s.l.. (PDF 394 kb) [file 12864_2016_3480_MOESM4_ESM.pdf]
