# Supplementary material for: Comparative analysis of the mitochondrial genomes of Colletotrichum gloeosporioides sensu lato: insights into the evolution of a fungal species complex interacting with diverse plants
Source: BMC Genomics. 2017 Feb 15;18:171. doi: 10.1186/s12864-016-3480-x (PMC5311727; doi:10.1186/s12864-016-3480-x)
Supplement: Additional file 5: Figure S4. — PCR amplification results of primers tested for differentiating C. gloeosporioides s.l. from C. actutatum s.l.. (PDF 1260 kb) [file 12864_2016_3480_MOESM5_ESM.pdf]

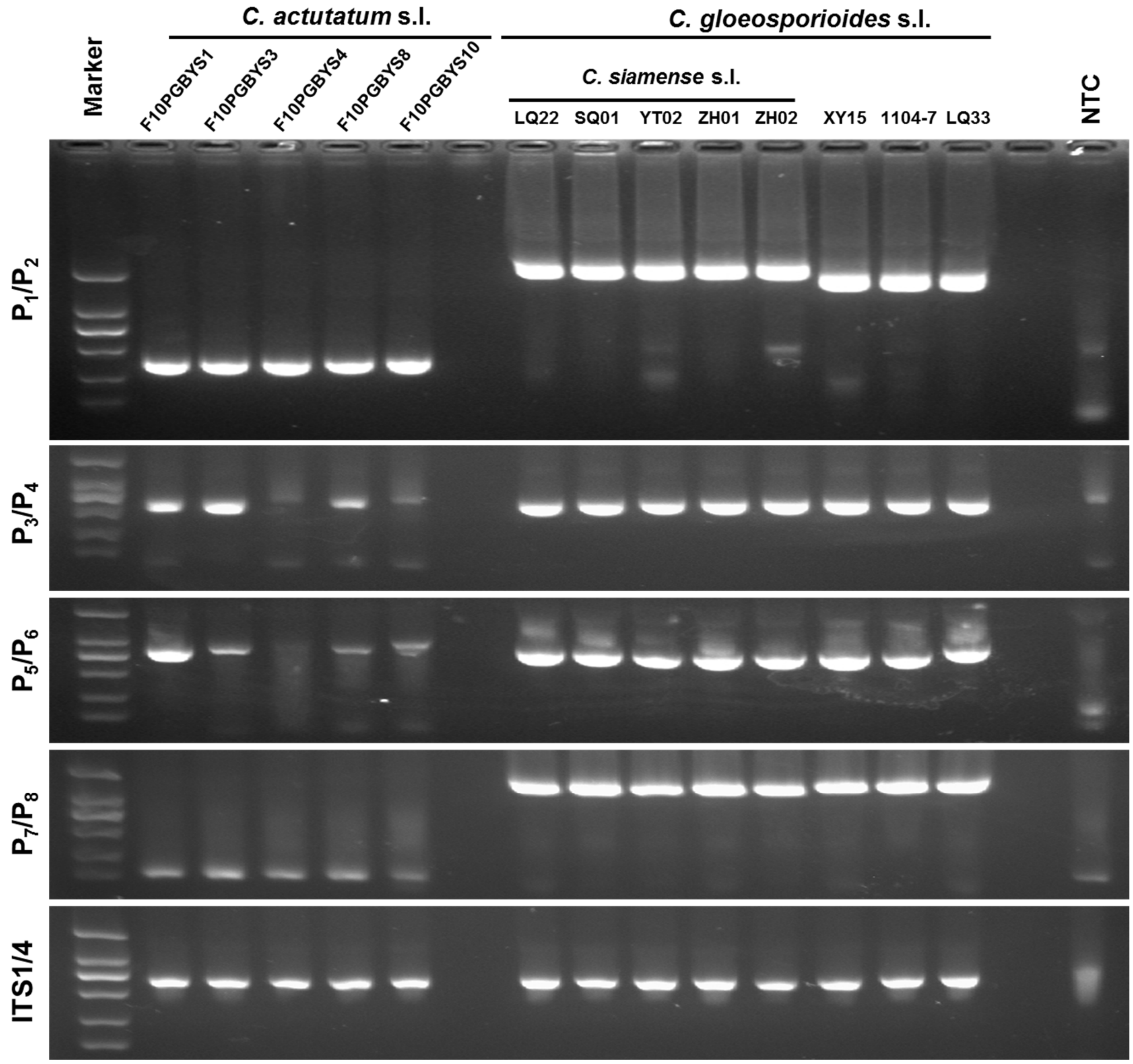

Figure S4. PCR amplification results of the primers tested for differentiating *C. gloeosporioides* s.l. from *C. actutatum* s.l.. ITS1/4 was used as a positive control. P_1/P_2 showed the best specificity and consistency among all tested ones. In P_1/P_2 result, the smaller amplicon sizes of XY15, 1104-7 and LQ33 were due to a smaller cob-1 intron size (Figure S4a). NTC, no template control.
