# Supplementary material for: Comparative analysis of the mitochondrial genomes of Colletotrichum gloeosporioides sensu lato: insights into the evolution of a fungal species complex interacting with diverse plants
Source: BMC Genomics. 2017 Feb 15;18:171. doi: 10.1186/s12864-016-3480-x (PMC5311727; doi:10.1186/s12864-016-3480-x)
Supplement: Additional file 6: Figure S5. — Maximum likelihood phylogenetic trees based on concatenated mitochondrial proteins and concatenated coding DNAs. (PDF 322 kb) [file 12864_2016_3480_MOESM6_ESM.pdf]

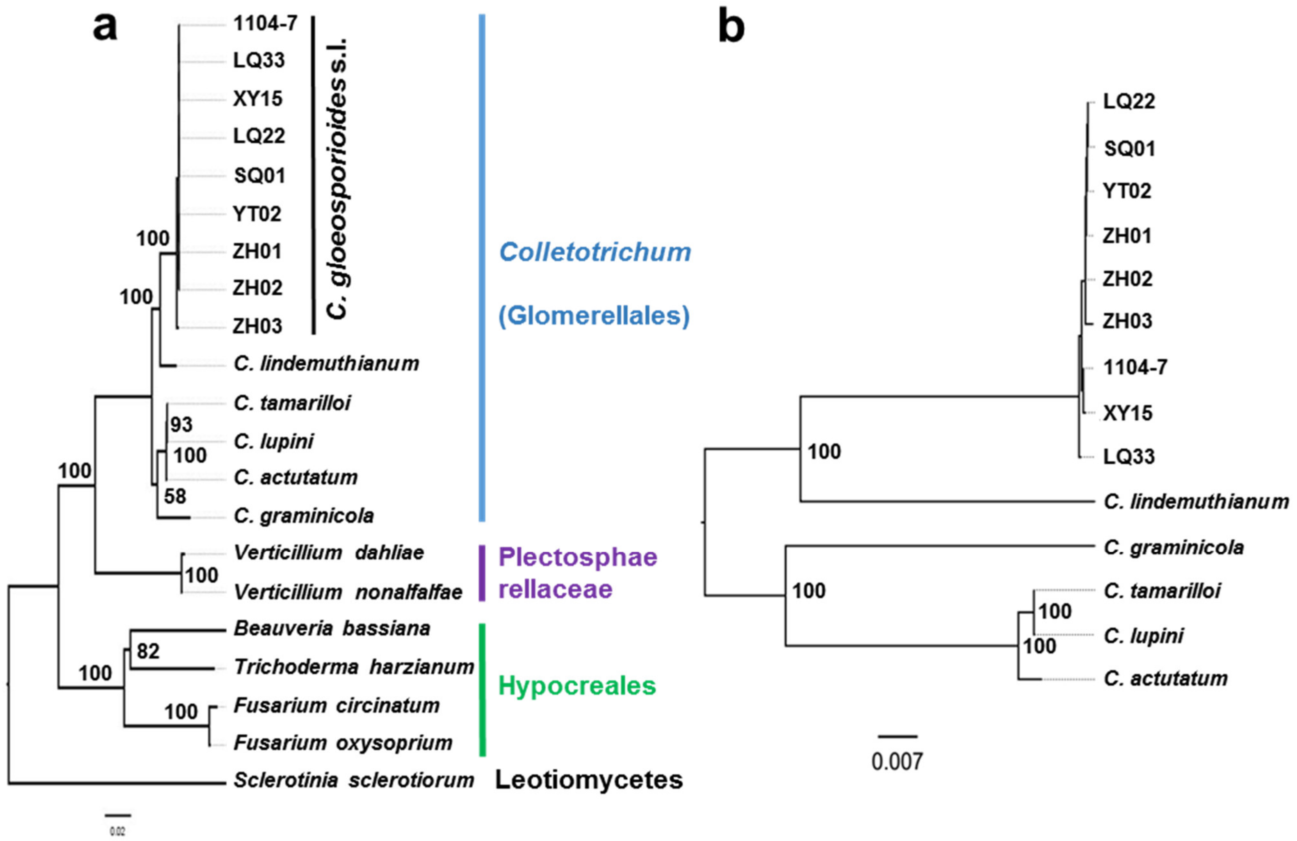

Figure S5. Maximum likelihood phylogenetic trees based on concatenated mitochondrial proteins and concatenated coding DNAs. **a.** The protein tree was constructed based on the unambiguously aligned portions of 13 proteins (Cob, Cox1, Cox2, Cox3, Nad1, Nad2, Nad3, Nad4, Nad5, Nad6, Atp6, Atp8, Atp9). The cpREV + F + I amino acid substitution model was used to generate the tree. GenBank accessions for *Colletotrichum* mitogenomes are listed in Additional file 1: Table S3. GenBank accessions for additional mitogenomes are as follows. *Verticillium dahliae*, DQ351941; *Verticillium nonalfalfae*, KR704425; *Fusarium circinatum*, JX910419; *Fusarium oxysporum*, KR952337; *Beauveria bassiana*, EU371503; *Metacordyceps chlamydosporia*, KF479445; *Trichoderma harzianum*, KR952346; *Sclerotinia sclerotiorum*, KT283062. **b.** The exon tree. Exon alignments of 15 genes (14 oxidative phosphorylation proteins and *rps1*) were concatenated for phylogenetic tree construction. The GTR + G + I substitution model was used. In **a** and **b**, numbers at the nodes are bootstrap values based on 1,000 replicates, certain bootstrap values in the *C. gloeosporioides* s.l. clade are omitted for easier visualization.
