# Supplementary material for: Comparative analysis of the mitochondrial genomes of Colletotrichum gloeosporioides sensu lato: insights into the evolution of a fungal species complex interacting with diverse plants
Source: BMC Genomics. 2017 Feb 15;18:171. doi: 10.1186/s12864-016-3480-x (PMC5311727; doi:10.1186/s12864-016-3480-x)
Supplement: Additional file 7: Figure S6. — Fifty percent majority rule consensus Bayesian trees based on different gene concatenation schemes. (PDF 475 kb) [file 12864_2016_3480_MOESM7_ESM.pdf]

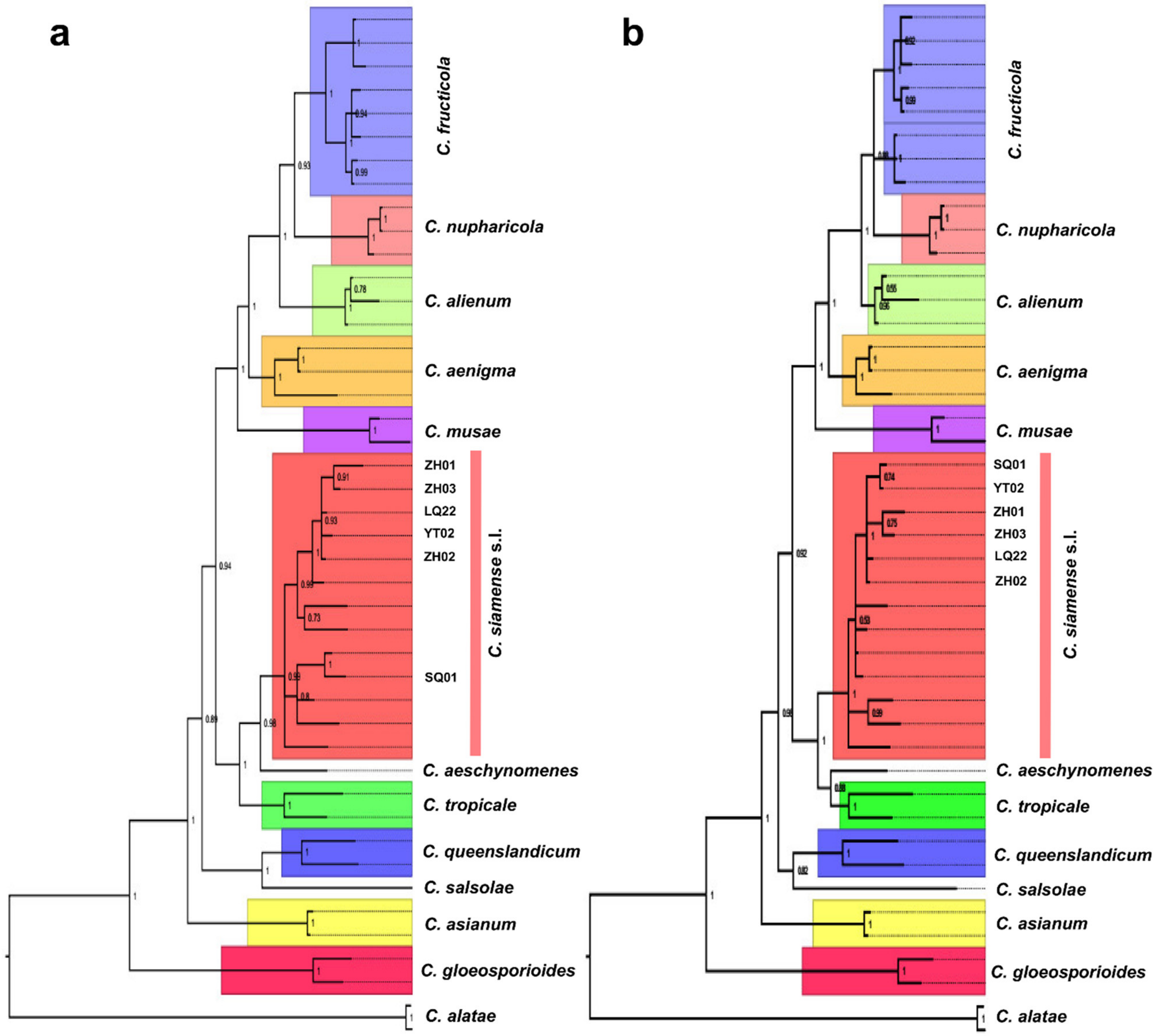

Figure S6. Fifty percent majority rule consensus Bayesian trees based on different gene concatenation schemes. **a.** tree constructed based on the concatenation of GADPH, CAL, SOD2, ACT, CHS, and TUB2. **b.** tree constructed based on the concatenation of GADPH, CAL, ACT, CHS, and TUB2. The trees were constructed in the same procedure as the one in Figure 1. The three tree topologies are highly similar. Yet by comparing the three tree topologies, it is clear that presence/absence of the GS and SOD2 markers strongly affects the phylogenetic positions of YT02 and SQ01.
