# Supplementary material for: Comparative analysis of the mitochondrial genomes of Colletotrichum gloeosporioides sensu lato: insights into the evolution of a fungal species complex interacting with diverse plants
Source: BMC Genomics. 2017 Feb 15;18:171. doi: 10.1186/s12864-016-3480-x (PMC5311727; doi:10.1186/s12864-016-3480-x)
Supplement: Additional file 8: Figure S7. — DNA polymorphism sites in the GS (glutamine synthetase) and SOD2 (manganese-superoxide dismutase) alignments of C. siamense s.l. isolates. (PDF 464 kb) [file 12864_2016_3480_MOESM8_ESM.pdf]

GS

	26	49	50	55	67	68	76	116	118	121	145	262	322	372	429	458	545	564	566	600	627	629	634	637	755	784	785	797	806	844	856
YT02	C	C	A	T	T	C	C	T	T	C	G	G	C	T	A	A	T	G	C	T	A	A	A	G	C	C	A	T	T	C	G
SQ01	A	C	T	C	C	T	G	T	C	C	A	G	T	T	G	G	C	A	T	C	A	G	C	A	A	T	G	C	C	T	C
LQ22	A	C	T	C	C	T	G	T	C	C	A	G	T	T	G	G	C	A	T	C	A	G	C	A	A	T	G	C	C	T	C
ZH01	C	–	T	C	T	T	C	G	C	T	A	G	T	A	G	A	T	G	C	C	T	G	A	G	C	T	G	C	C	C	C
ZH02	C	–	T	C	T	T	C	G	C	T	A	G	T	A	G	A	T	G	C	C	T	G	A	G	C	T	G	C	C	C	C
ZH03	A	C	T	C	T	T	C	T	C	C	A	A	T	T	G	G	C	G	T	C	A	G	C	A	A	T	G	C	C	T	C
YT02-specific mutation?			*	*		*			*		*		*		*					*		*				*	*	*	*		*

SOD2

	23	65	83	95	101	146	216	217	219	221	224	239	255	281	305	368
SQ01	G	C	A	C	C	T	G	A	C	C	C	A	T	C	A	C
YT02	A	C	G	T	T	C	A	G	C	G	T	T	C	A	C	T
LQ22	A	C	G	T	T	C	A	G	C	G	T	T	C	A	C	T
ZH01	A	C	G	T	T	C	A	G	G	G	T	T	T	C	C	T
ZH02	A	T	G	T	T	C	A	G	C	G	T	T	C	A	C	T
ZH03	A	C	G	T	T	C	A	G	C	G	T	T	C	A	C	T
Codon position	3	3	3	3	3	3	1	2	1	3	3	3	1	3	3	3
Synonymous or nonsynonymous?	S	S	S	S	S	S	N	N	N	S	S	S	S	S	S	S
SQ01-specific mutation?	*		*	*	*	*	*	*		*	*	*			*	*

Figure S7. DNA polymorphism sites in the GS (glutamine synthetase) and SOD2 (manganese-superoxide dismutase) alignments of *C. siamense* s.l. isolates. The 858 bp long GS alignment is made up of a long partial intron (1-850 bp) and a short partial coding region (851-858 bp), the 374 bp long SOD2 alignment is entirely in the coding region.
